# Task‐based functional connectivity in aging: How task and connectivity methodology affect discovery of age effects

**DOI:** 10.1002/brb3.1954

**Published:** 2020-11-18

**Authors:** Eleanna Varangis, Christian G. Habeck, Yaakov Stern

**Affiliations:** ^1^ Cognitive Neuroscience Division Department of Neurology College of Physicians and Surgeons Columbia University New York NY USA

**Keywords:** aging, cognition, fMRI, functional connectivity

## Abstract

**Introduction:**

Past studies have found that healthy aging has a significant effect on the organization and function of networks in the human brain. Many of these studies have examined how functional connectivity during one task or at rest is affected by aging; however, few studies have systematically examined how the effect of age on functional connectivity may vary as a function of choice of in‐scanner task.

**Methods:**

The present study included healthy adults between the ages of 20 and 80 and examined a variety of metrics of functional connectivity during performance of 11 in‐scanner tasks, falling into 4 cognitive domains: vocabulary, processing speed, fluid reasoning, and episodic memory. Functional connectivity was assessed at three levels: average correlations within and between 10 networks, system segregation (sensorimotor vs. association networks), and whole‐brain graph theory metrics (global efficiency and modularity).

**Results:**

Results showed that the effect of age on these metrics differed as a function of task—for example, age had a more consistent effect on functional connectivity metrics computed during fluid reasoning tasks; however, there was less of an effect of age on functional connectivity metrics computed during tasks of episodic memory. Further, some of these measures showed relationships with behavioral performance on the in‐scanner task, with different networks playing a role in the different cognitive domains.

**Conclusion:**

These findings suggest that while aging may be generally associated with reductions in within‐ and between‐network connectivity, system segregation, global efficiency, and modularity, the magnitude and presence of these effects varies by in‐scanner task.

## Graphical abstract

The present study examined the effects of task domain and age on functional connectivity computed during a set of 11 cognitive tasks. Results showed that task domain had a significant effect on the presence and magnitude of the effect of age on functional connectivity measures, and that age‐related connectivity differences were more readily observed during tasks of fluid reasoning, and less likely to be observed during memory tasks. These findings suggest that task choice has a significant effect on estimation of age‐related differences in neurocognitive functioning.

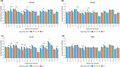

## 1 INTRODUCTION

Studies investigating the effect of aging on functional neural recruitment during a cognitive task have found systematic differences in the way older and younger adults utilize specific regions of the brain when performing a task, along with relationships between this differential utilization and task performance. Current trends in the field of neuroimaging research have expanded the scope of task‐based fMRI analyses to also probe differences in the functional connectivity between brain regions as a function of task performance and aging. Past studies have shown that normal aging is associated with alterations in functional connectivity at rest, suggesting that even in the absence of a cognitive challenge, older age is associated with differences in functional network architecture and function. These studies have largely shown that aging is associated with reductions in within‐network connectivity accompanied by increases in between‐network connectivity, resulting in a less modular/segregated brain (Betzel et al., 2014; Chan et al., 2014; Geerligs, Renken, et al., 2015; Iordan et al., 2017). Some of these studies have even found links between these patterns of connectivity at rest and cognitive performance outside of the scanner, suggesting a potential connectivity‐based mechanism underlying some age‐related differences in cognitive performance (Chan et al., 2014; Geerligs, Renken, et al., 2015; King et al., 2017; Onoda et al., 2012; Sala‐Llonch et al., 2014; Zonneveld et al., 2019).

Some recent studies have expanded this line of investigation into the ways in which functional connectivity during performance of a cognitive task is affected by age. Initial studies on the effect of older age on functional connectivity during a task found that, unlike younger adults, older adults show attenuation of negative correlations between regions in task‐relevant and task‐irrelevant networks (Sala‐Llonch et al., 2012) and that the degree to which older adults showed a negative correlation between these networks is related to performance on executive function/working memory tasks (Miller et al., 2008; Prakash et al., 2012). While these studies focused on specific connections and regions of interest (ROIs), later studies have expanded this investigation to include connections across the whole brain, either using analyses identifying regions showing significantly correlated blood oxygen level‐dependent (BOLD) fluctuations with fluctuation in a predefined seed (or set of seeds; Andrews‐Hanna et al., 2007; Burianova et al., 2015; Campbell et al., 2012; Spreng et al., 2016; Wang et al., 2010); using clustering to identify functional networks from whole‐brain BOLD data (Archer et al., 2016; Geerligs et al., 2014); or using predefined anatomical or spherical ROIs to examine BOLD time series correlations among these ROIs (Geerligs, Rubinov, et al., 2015). Consistently, these studies have found age‐related alterations in task‐based functional connectivity, with some finding interactions between age and scan type (task, rest, etc.) or task load on functional connectivity metrics (Archer et al., 2016; Burianova et al., 2015; Geerligs, Rubinov, et al., 2015).

The observation that age affects connectivity differentially based on scan type or cognitive load has considerable implications for the study of cognitive aging. Determining which aspects of connectivity and what scan conditions are most sensitive to age or clinical status is critical in assessing the clinical utility of these measures. Thus, studies examining connectivity across multiple scan conditions are critical in determining what aspects of functional connectivity are most sensitive to aging. In this vein, a few studies have compared connectivity metrics across both rest and different task conditions in order to see how cognitive state affects these connectivity patterns. Two studies found that task condition had a significant effect on the connectivity patterns observed—Archer et al. (2016) found a more widespread effect of age on task‐based, compared to resting state, connectivity, and Geerligs, Renken, et al. (2015) found that patterns of connectivity differences by age group differed based on the scan condition, such that subcortical networks were more sensitive to age at rest, while association networks were more sensitive to age during a sensorimotor task. Thus, in both studies, scan condition (task vs. rest or task vs. task) had a significant effect on the magnitude and location of the effect of participant age on functional connectivity within and between networks in the brain.

While these studies have examined the effect of age on functional connectivity during one or two in‐scanner tasks, one past study from our group directly compared patterns of functional connectivity during cognitive tasks that corresponded to four cognitive domains (vocabulary, processing speed, fluid reasoning, and episodic memory) in the same set of participants (Varangis, Razlighi, et al., 2019). Using a novel latent factor modeling approach, this study specifically examined the effects of age and task domain on functional connectivity between 6 latent cognitive networks (default mode network, or DMN; frontoparietal network, or FP; cingulo‐opercular network, or CO; salience network, or Sal; dorsal attention network, or DAN; and memory network, or Mem). Results showed that task domain and age group (younger adults, middle‐aged adults, and older adults) had independent effects on between‐factor connectivity, but that age did not modify the effect of task domain on between‐factor connectivity. While this study provided evidence to suggest that both task and age affect connectivity patterns between networks and that some of these patterns were related to performance on these in‐scanner tasks, it did not comprehensively examine multiple aspects of functional connectivity to evaluate whether more commonly utilized metrics of functional connectivity (i.e., graph theory, average correlation) show similar effects of both age and task. Additionally, due to the nature of the latent factor modeling, only between‐factor connectivity was examined, limiting comparison with studies focusing on within‐network connectivity. Further, one benefit of the latent factor approach was that it allowed ROI time series to freely load on network factors at the participant level; however, this makes comparisons with other studies using more standard network‐based approaches (e.g., average correlation between one network and another network) difficult. Past studies assessing the effect of aging on a variety of metrics of functional connectivity at rest have found that age effects are not ubiquitous and that they may only emerge when using specific metrics, in specific networks, or at a specific range of thresholds (Geerligs, Renken, et al., 2015; Iordan et al., 2017; Song et al., 2014; Varangis, Habeck, et al., 2019). In order to facilitate qualitative comparison of the effects of age on task‐ vs. resting state connectivity, the present study followed a similar analytic approach to that used previously on resting state fMRI scans by our group to examine the effect of participant age on multiple varied approaches to characterizing whole‐brain functional connectivity (Varangis, Habeck, et al., 2019). Thus, extending this line of research to assess how these metrics may be affected by age during a cognitive task, as well as how these patterns may differ based on the cognitive task being performed, is a logical next step in characterizing the full extent and magnitude of the effects of age on functional connectivity across the whole brain.

The present study aimed to more comprehensively examine the effects of aging on task‐based functional connectivity using several different whole‐brain functional connectivity methods (average correlation within/between networks, system segregation, global efficiency, and modularity), and across four different cognitive domains (vocabulary, perceptual speed, fluid reasoning, and episodic memory). Our previous study investigated functional connectivity between latent network factors in this sample; however, the present study expands upon this study by: (a) including a substantially larger sample of participants (many were excluded from the previous study due to poor latent factor model fit); (b) utilizing more standard, widely used, connectivity metrics to facilitate comparison with results of past studies; (c) including noncognitive, somatosensory (somatomotor hand, somatomotor mouth, auditory, and visual), networks in addition to cognitive networks (DMN, FP, CO, Sal, DAN, and the ventral attention network, or VAN); and (d) examining several different features of both network‐based and whole‐brain connectivity. Based on past studies assessing the effects of aging on functional connectivity during rest and task scans, the hypotheses of the present study are as follows: (1) older adults will show weaker within‐network connectivity, stronger between‐network connectivity, and reduced system segregation across all tasks; (2) there will be more networks that show an effect of age on within/between‐network connectivity during fluid reasoning and processing speed tasks, and fewer showing an effect of age during vocabulary and memory tasks; and (3) whole‐brain graph theory metrics of connectivity will show some modulation by both age and cognitive task, with differences between age groups more readily observed during fluid reasoning and processing speed tasks. Further, the present study will present some exploratory data examining relationships between functional connectivity metrics and in‐scanner task performance in order to get a better sense of which metrics and networks may play a role in cognitive performance.

In addition to expanding upon existing research on age modulation of task‐based functional connectivity, the present study will also examine the effect of age on task‐based connectivity that is computed based on a few different common connectivity methodologies, ranging from highly network‐specific techniques (network‐based correlations) to whole‐brain techniques (global efficiency). While every metric included in the present study has previously been found to be affected by age either at rest or during a cognitive task in past studies, the present study will incorporate and compare across all metrics within the same participant sample. Given the challenge of completing a cognitive task and the cognitive functions ascribed to several of the networks included in the present analyses, it is expected that network‐based metrics may be particularly sensitive to age effects on functional connectivity during a task (e.g., Geerligs, Renken, et al., 2015). This may be particularly evident during tasks that draw more heavily on executive function resources, as these tasks may necessitate higher levels of network integration/disintegration (i.e., Varangis, Razlighi, et al., 2019). Additionally, metrics computed based on network parcellation (i.e., system segregation and modularity) may also show age modulation of task effects due to their inclusion of network properties during computation (either based on predefined networks as in system segregation or based on individually detected networks as in modularity). However, whole‐brain metrics that are agnostic to network membership/ROI localization, such as global efficiency, may be less likely to show robust effects of participant age or task effects.

## 2 METHODS

### 2.1 Sample

The sample for the present study was comprised of participants who completed the baseline visit for the Reference Ability Neural Network (RANN) study (*N* = 396; Stern et al., 2014). All participants were native English speakers, right‐handed, free of MRI contraindications, and read at a fourth grade reading level or above. Screening was performed prior to enrollment in order to ensure that no participants had any psychological or medical conditions that could affect cognitive function and that older adults did not meet criteria for dementia or MCI at baseline. Based on age grouping used for display purposes in a previous study (Chan et al., 2014), participants were divided into four different age groups to facilitate testing of moderation by age: younger adults (YA; age 20–34, *n* = 90), younger middle‐aged adults (yMA; age 35–49, *n* = 64), older middle‐aged adults (oMA; age 50–64, *n* = 113), and older adults (OA; age 65–80, *n* = 129). For the present analyses, the following additional criteria were established: completion of all 11 in‐scanner tasks (*N* = 335; YA *n* = 75, yMA *n* = 64, oMA *n* = 97, OA *n* = 99), and less than 30% motion artifact data removal (scrubbing; Parkes et al., 2018; Power et al., 2012) within each of the four domain time series (*N* = 302; YA *n* = 72, yMA *n* = 60, oMA *n* = 86, OA *n* = 84; see Table 1).

**TABLE 1:** Participant demographics

	YA (*n* = 72)	yMA (*n* = 60)	oMA (*n* = 86)	OA (*n* = 84)	*p*‐Value of difference
Age	28.292 (4.026)	42.333 (4.425)	57.477 (4.516)	71.190 (4.135)	<.001
Gender (%F)	68.06%	48.33%	48.84%	48.81%	.277
Education	16.153 (2.311)	15.867 (2.554)	16.035 (2.072)	16.464 (2.613)	.476
VOCAB	−0.143 (0.880)	−0.161 (0.825)	0.029 (0.880)	0.099 (0.767)	.201
SPEED	−0.359 (0.810)	−0.178 (0.696)	−0.049 (0.731)	0.300 (0.730)	<.001
FLUID	0.280 (0.889)	0.001 (0.759)	−0.054 (0.884)	−0.088 (0.927)	.065
MEM	0.268 (0.811)	0.054 (0.632)	−0.047 (0.694)	−0.193 (0.815)	.004

Note

Values reflect group means (standard deviations in parentheses), and percentage female within each age group. *p*‐Values reflect *p*‐values for one‐way ANOVAs for all continuous variables, or chi‐square test for Gender.

Abbreviations: FLUID, average z‐score of performance on three in‐scanner fluid reasoning tasks; MEM, average z‐score of performance on three in‐scanner Episodic Memory tasks; OA, older adult; oMA, older middle‐aged adult; SPEED, average z‐score of performance on three in‐scanner processing speed tasks; VOCAB, average z‐score of performance on three in‐scanner Vocabulary tasks; YA, younger adult; yMA, younger middle‐aged adult.

### 2.2 In‐scanner cognitive tasks

The cognitive outcome measures included in the present analyses are represented by composite z‐scores of performance on tasks completed during the fMRI scan. The in‐scanner tasks included 11 cognitive tasks that were previously found to cluster into four primary reference abilities (Salthouse, 2009; Salthouse & Ferrer‐Caja, 2003): vocabulary (VOCAB: synonyms and antonyms), perceptual speed (SPEED: digit symbol, letter comparison, pattern comparison), fluid reasoning (FLUID: paper folding, matrix reasoning, and letter sets), and episodic memory (MEM: logical memory, word order, and paired associates). These tasks have been used extensively in previous studies conducted by the authors of the current study; for detailed information on these tasks, please see (Razlighi et al., 2017; Stern et al., 2014; Varangis, Razlighi, et al., 2019). One task, the Picture Naming task from the VOCAB reference ability, was not included in the present analyses due to in‐scanner motion arising from participants speaking their responses aloud during the scanned task. Performance on each individual task was z‐scored; then, domain‐based z‐scores were generated by averaging the z‐scores of the tasks within each domain. For VOCAB, FLUID, and MEM tasks, higher z‐scores mean higher accuracy on the in‐scanner task; for SPEED tasks, z‐scores were reverse coded such that higher z‐scores mean faster reaction times.

### 2.3 fMRI scan parameters

The present study collected fMRI scans during the in‐scanner tasks mentioned above. All participants completed these scans on a 3.0T Philips Achieva Magnet over the course of two 2‐hr MR imaging sessions. T1‐weighted images of the whole brain were acquired for each subject with a Magnetization Prepared Rapid Gradient Echo (MPRAGE) sequence with the following parameters: TE/TR: 3/6.5 ms; Field of view: 256 mm; Flip angle: 8°; In‐plane resolution: 256 × 256 voxels; Slice thickness/gap: 1/0 mm; Slices: 180. fMRI blood oxygen level‐dependent (BOLD) scans were collected during each of the 11 in‐scanner tasks mentioned above with the following parameters: TE/TR: 20/2,000 ms; Field of view: 240 mm; Flip angle: 72°; In‐plane resolution: 112 × 112 voxels; Slice thickness/gap: 3/0 mm; Slices: 41.

### 2.4 fMRI data processing

Images were preprocessed using an in‐house developed native space method (Razlighi, et al., 2014) as described and utilized previously in Varangis, Razlighi, et al. (2019). The preprocessing pipeline included slice‐timing correction and motion correction performed in FSL (Jenkinson, Bannister, et al., 2002; Jenkinson, Beckmann, et al., 2012), calculation of frame‐wise displacement (FWD; as described in Power et al., 2012), volume replacement for contaminated volumes (Carp, 2013; Power et al., 2012), band‐pass filtering using flsmaths–bptf (Jenkinson, Beckmann, et al., 2012), and residualization of the processed data with respect to FWD, root mean square difference of the BOLD signal, left and right hemisphere white matter, and lateral ventricular signals (Birn, et al., 2006). T1 image segmentation was performed using FreeSurfer (Dale, et al., 1999, Fischl, Salat, et al., 2002, Fischl, van der Kouwe, et al., 2004) and inspected visually for any possible inaccuracies. In order to perform the functional connectivity analyses described below, the coordinates of the 264 ROIs identified by Power and colleagues (2011) were transferred to native space via nonlinear registration of the subject's structural scan to the MNI template using the ANTS software package. Next, a 10 mm radius spherical mask was generated for each coordinate and intersected with the FreeSurfer gray matter mask in order to derive the gray matter‐registered ROI masks for each of the 264 ROIs. An intermodal, intrasubject, rigid‐body registration of the fMRI reference image and T1 scan was then performed using FLIRT with 6 degrees of freedom, normalized mutual information as the cost function (Jenkinson & Smith, 2001), in order to transfer ROI masks from T1 space to fMRI space. These transferred ROI masks were used to average all voxels within each mask to obtain a single fMRI time series for each of the 264 ROIs.

Time series data were then concatenated by domain, yielding 4 sets of time series data that were modeled separately as blocked designs: VOCAB (concatenated synonyms and antonyms data; 388 volumes, SPEED (concatenated digit symbol, letter comparison, and pattern comparison data; 595 volumes), FLUID (concatenated paper folding, matrix reasoning, and letter sets data; 1,290 volumes), and MEM (concatenated logical memory, word order, and paired associates data; 517 volumes). In order to remove any unique task‐specific variance from the domain time series, task was regressed out of each ROI’s time series. These residualized time series were then used to generate correlation matrices among all ROIs (264 ROIs by 264 ROIs). The diagonal of each correlation matrix was set to zero for all graph theory analyses, and “NA” for all average correlation analyses, in order to remove correlations between an area and itself from analyses. Further, as per the utilization recommendations for this parcellation scheme (Power et al., 2011), all ROIs with centers within 20 mm of one another in standard space were excluded from all analyses. ROIs were then labeled based on the Power (2011) network assignments, with the following networks being selected for analysis based on their inclusion in similar past studies (Chan et al., 2014; Geerligs, Renken, et al., 2015): visual (Vis; 31 ROIs), somatomotor mouth (Mouth; 5 ROIs), somatomotor hand (Hand; 30 ROIs), auditory (Aud; 13 ROIs), default mode (DMN; 58 ROIs), salience (Sal; 18 ROIs), cingulo‐opercular (CO; 14 ROIs), frontoparietal (FP; 25 ROIs), dorsal attention (DAN; 11 ROIs), and ventral attention (VAN; 9 ROIs).

### 2.5 Functional connectivity analyses

Individual correlation matrices were used to compute several measures of functional connectivity.

#### 2.5.1 Positive/negative correlation weights

Average positive and negative correlation were computed within and between all networks of interest. Within‐network correlations were characterized as those reflecting correlations between ROIs within a specific network; between‐network correlations were characterized as those reflecting correlations between ROIs from one network and those of all other networks. Average positive correlation was computed by setting all negative correlation values to “NA,” then taking the average within‐ and between‐network positive correlation for each network. Average negative correlation was computed by setting all positive correlation values to “NA,” then taking the average within‐ and between‐network negative correlation for each network. Due to few negative within‐network correlations (and concern as to how to interpret these values), only between‐network negative correlations were included in the analysis of negative correlations. Thus, data from this analysis included the average within‐network positive correlation (10 values), average between‐network positive correlation (10 values), and average between‐network negative correlation (10 values) for each participant. In order to examine the effect of age and domain on positive correlation strength, a 4 (Domain: VOCAB, SPEED, FLUID, MEM) x 4 (age group: YA, yMA, oMA, OA) × 2 (correlation direction: within, between) × 10 (network: Vis, Mouth, Hand, Aud, DMN, Sal, FP, CO, DAN, VAN) MANCOVA (covariate: scrubbing percentage) was performed. For this analysis only, the sample size was reduced by 20 participants to 282 participants (YA *n* = 65, yMA *n* = 54, oMA *n* = 85, OA *n* = 78) since not all participants had positive within‐network correlations in some smaller networks (i.e., VAN); as such, the remaining 282 participants had valid estimates for average positive within‐ and between‐network correlations in all 10 networks. To examine the effect of age and domain on negative correlation strength, a 4 (Domain: VOCAB, SPEED, FLUID, MEM) x 4 (age group: YA, yMA, oMA, OA) × 10 (network: Vis, Mouth, Hand, Aud, DMN, Sal, FP, CO, DAN, VAN) MANCOVA (covariate: scrubbing percentage) was performed. Significant interactions were probed using follow‐up MANCOVA and ANOVA analyses.

#### 2.5.2 System segregation

These data were also used to derive the metric of system segregation introduced by Wig and colleagues (Chan et al., 2014; Chan, et al., 2017; Wig, 2017). This metric reflects the degree to which the brain segments into networks (or systems) that function independently of one another—high values reflect greater functional separation between networks, while lower values reflect less functional separation between networks. In order to compute this metric, only positive correlations among the ten networks identified above were considered, and all negative correlations were set to zero (Chan et al., 2014; Chan, et al., 2017). As in previous uses of this metric, system segregation was computed separately across all somatomotor networks (auditory, visual, somatomotor hand, and somatomotor mouth) and across all association networks (default mode, frontoparietal, cingulo‐opercular, ventral attention, dorsal attention, and salience). Then, the average within‐ and between‐network correlations were calculated for each type of network separately (average correlation within and between all somatomotor networks, excluding association networks; average correlation within and between all association networks, excluding somatomotor networks), and the system segregation was defined as:

$$\mathrm{SS}=\frac{{\overset{‐}{z}}_{\mathrm{within}}‐{\overset{‐}{z}}_{\mathrm{between}}}{{\overset{‐}{z}}_{\mathrm{within}}}$$

A 4 (Domain: VOCAB, SPEED, FLUID, MEM) × 2 (Networks: somatomotor networks versus. association networks) × 4 (Age Group: YA, yMA, oMA, OA) MANCOVA (covariate: scrubbing percentage) was used to test the effects of age and domain on this metric.

#### 2.5.3 Graph theory metrics of global connectivity

Two graph theory metrics of functional connectivity were also computed using the Brain Connectivity Toolbox (Rubinov & Sporns, 2010) (www.brain‐connectivity‐toolbox.net) in order to measure additional aspects of global and nodal connectivity. Global connectivity was assessed using the graph theory metrics of modularity (the extent to which the correlation matrix can be partitioned into networks that maximize within‐group connections and minimize between‐group connections; Newman, 2006) and global efficiency (average inverse shortest path length; Latora & Marchiori, 2001). In order to ensure that results were not biased by the connectivity weight threshold applied to the correlation matrices, a range of thresholds between 2% and 10% (in increments of 1%) were applied to matrices during computation of each metric (i.e., Geerligs, Renken, et al., 2015). Based on this thresholding, all graph theory metrics were only computed on positive correlation weights, with all correlations not meeting this threshold being set to zero. As such, all graph theory analyses evaluate the effects of both age group and threshold on the metric of interest. In order to assess the effect of age and domain on these graph theory metrics, a 4 (Domain: VOCAB, SPEED, FLUID, MEM) × 4 (age group: YA, yMA, oMA, OA) × 9 (threshold: 2%–10%) MANCOVA (covariate: scrubbing percentage) was conducted for each metric. Significant interactions were probed using follow‐up MANCOVA (age group × network at each threshold) and one‐way ANOVA (effect of age group on each network for thresholds exhibiting a significant age × network interaction) analyses.

Due to the nature of the hypotheses being tested in the present set of analyses, only results relating to the effects of participant age on the set of connectivity metrics presented above will be discussed in detail in the context of the present manuscript. All outcomes of statistical tests will be presented in referenced supplementary text, tables, and figures; however, interpretation and discussion will be limited to those metrics showing effects of age, or interactions between age and other factors (i.e., functional network, correlation direction, task domain, and matrix threshold).

### 2.6 Correlational brain–behavior analyses

In order to probe which of these metrics may be related to performance on the in‐scanner task, Pearson partial correlational analyses were conducted between functional connectivity metrics computed during each task and performance on the in‐scanner tasks, controlling for years of education and gender. Further, in order to account for the effect of participant age on these relationships, an additional set of Pearson partial correlations were computed with respect to years of education, gender, and participant age. Due to the high number of functional connectivity metrics being computed for each task, and due to the relatively exploratory nature of these analyses, no p‐value correction was performed; however, interpretation is limited to those metrics and networks showing consistent relationships with task performance.

### 2.7 Ethics statement

The Columbia University Institutional Review Board approved all study procedures, and all participants provided written informed consent prior to participation.

## 3 RESULTS

### 3.1 Positive/negative correlation weights

**FIGURE 1:**
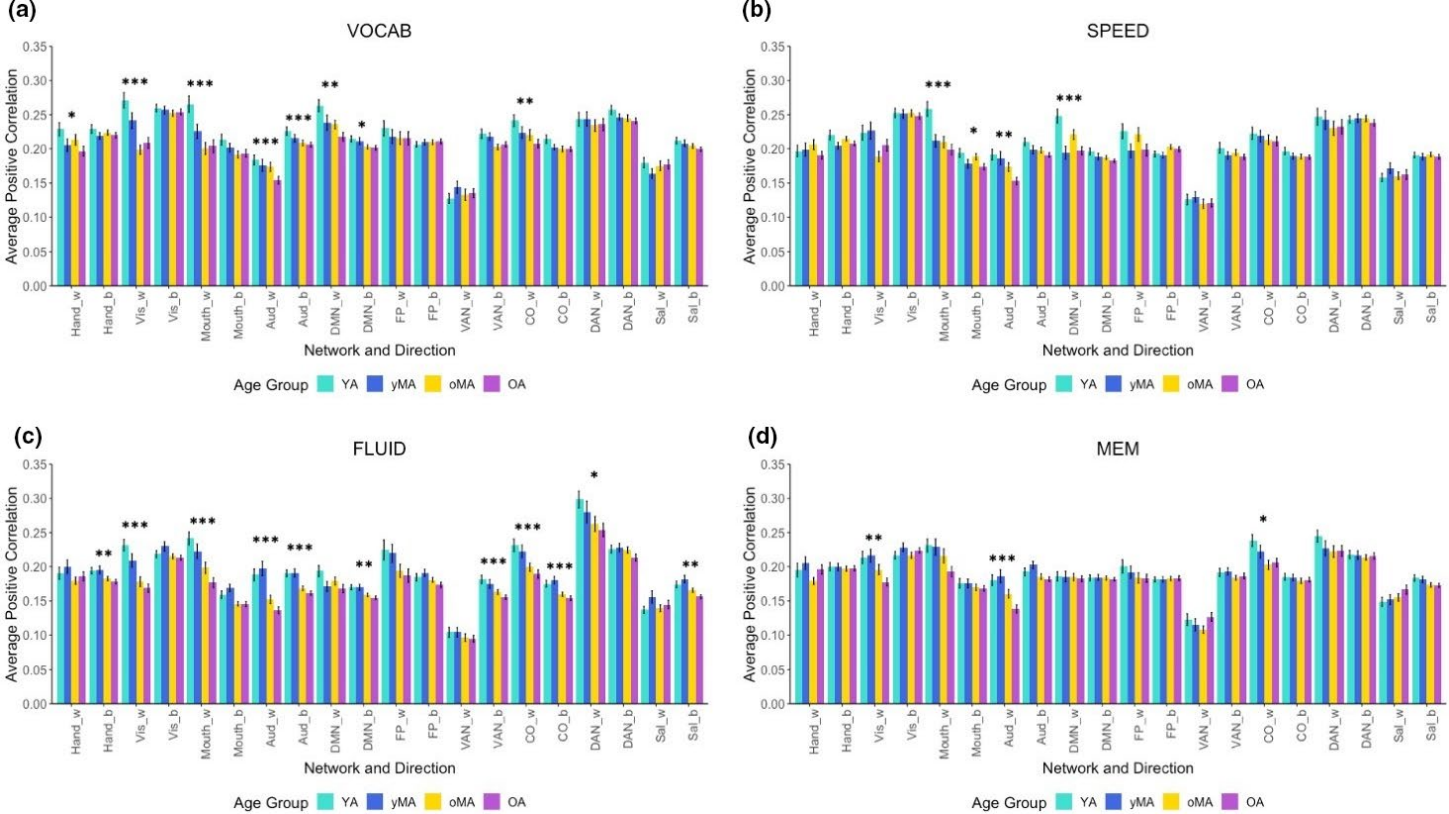
Average within(w)/between(b)‐network positive correlation for each age group, depicted for each task domain. Asterisks reflect significance of the difference between younger (YA) and older (OA) adults at each network (*p < .05, **p < .01, ***p < .001; all Bonferroni corrected within each domain). Networks are presented using acronyms defined in the text: Aud, Auditory; CO, Cingulo‐Opercular; DAN, Dorsal Attention Network; DMN, Default Mode Network; FP, Frontoparietal; Hand, Somatomotor Hand; Mouth, Somatomotor Mouth; Sal, Salience; VAN, Ventral Attention Network; Vis, Visual

A 4 (Domain: VOCAB, SPEED, FLUID, MEM) × 10 (Network) × 2 (Correlation direction: within, between) × 4 (Age Group: YA, yMA, oMA, OA) revealed a significant main effect of age group (*F*_3,274_ = 11.551, *p *< .001) on positive correlation strength. Further, there were significant interactions among domain and age group (*F*_9,822_ = 2.185, *p* = .021), network and age group (*F*_27,2,466_ = 2.552, *p *< .001), direction and age group (*F*_3,274_ = 13.859, *p *< .001), domain and network and age group (*F*_81,7,398_ = 1.503, *p* = .002), network and direction and age group (*F*_27,2,466_ = 2.670, *p *< .001), and domain and network and direction and age group (*F*_81,7,398_ = 1.575, *p* = .001). The interaction among domain, direction, and age group was not significant (*F*_9,822_ = 0.386, *p* = .942). The main effect of age suggested that, overall, older age was associated with lower positive correlation strength (see Table 2, Tables S1–S4). Follow‐up analyses probing the 4‐way interaction showed that age had a dampening effect on positive correlation strength for differing networks depending upon the domain being analyzed (see Figure 1, Table 2, Tables S1–S4). The auditory network showed an effect of age on within‐network correlations across all four domains, and however, other networks only showed an effect of age on within‐ or between‐network correlation strength in specific domains (i.e., Hand, CO, and Sal between‐network correlation strength only showed an effect of age during FLUID tasks).

**TABLE 2:** Post hoc test results for the interaction among age group, domain, network, and direction for both positive and negative correlations

	VOCAB		SPEED		FLUID		MEM	
	Positive	Negative	Positive	Negative	Positive	Negative	Positive	Negative
3‐way MANCOVA by Domain								
Network	**74.393 (<.001)**	n/a	**76.603 (<.001)**	n/a	**124.153 (<.001)**	n/a	**64.503 (<.001)**	n/a
Direction	**48.128 (<.001)**	n/a	**22.305 (<.001)**	n/a	**27.045 (<.001)**	n/a	1.96 (.163)	n/a
Age	**7.555 (<.001)**	n/a	**3.39 (.018)**	n/a	**15.221 (<.001)**	n/a	**4.392 (.005)**	n/a
Network*Age	**2.424 (<.001)**	n/a	**2.522 (<.001)**	n/a	**1.915 (.003)**	n/a	**1.897 (.004)**	n/a
Direction*Age	**7.373 (<.001)**	n/a	**7.359 (<.001)**	n/a	**9.078 (<.001)**	n/a	**4.759 (.003)**	n/a
Network*Direction	**49.332 (<.001)**	n/a	**44.542 (<.001)**	n/a	**61.134 (<.001)**	n/a	**54.392 (<.001)**	n/a
3‐way Interaction	**2.509 (<.001)**	n/a	**1.979 (.002)**	n/a	**2.336 (<.001)**	n/a	**2.292 (<.001)**	n/a
2‐way MANOVA by Domain and Direction								
Within‐network								
Network	**56.694 (<.001)**	n/a	**47.342 (<.001)**	n/a	**89.045 (<.001)**	n/a	**56.833 (<.001)**	n/a
Age	**11.210 (<.001)**	n/a	**6.428 (<.001)**	n/a	**19.368 (<.001)**	n/a	**7.549 (<.001)**	n/a
Network*Age	**2.590 (<.001)**	n/a	**2.259 (<.001)**	n/a	**2.070 (.001)**	n/a	**2.164 (<.001)**	n/a
Between‐network								
Network	**103.274 (<.001)**	**58.656 (<.001)**	**155.051 (<.001)**	**70.070 (<.001)**	**141.248 (<.001)**	**32.807 (<.001)**	**82.075 (<.001)**	**14.908 (<.001)**
Age	**3.517 (.016)**	**3.082 (.028)**	0.888 (.448)	**6.080 (<.001)**	**9.059 (<.001)**	**6.982 (<.001)**	1.309 (.272)	0.441 (.724)
Network*Age	**1.719 (.012)**	**1.593 (.027)**	**2.444 (<.001)**	**3.377 (<.001)**	**2.712 (<.001)**	1.295 (.141)	1.444 (.065)	0.810 (.743)
1‐way post hoc ANOVAs								
Within‐network								
Hand	**3.363 (.019)**	n/a	0.650 (.583)	n/a	1.657 (.176)	n/a	2.057 (.106)	n/a
Vis	**15.685 (<.001)**	n/a	**4.179 (.006)**	n/a	**17.904 (<.001)**	n/a	**6.070 (.001)**	n/a
Mouth	**10.444 (<.001)**	n/a	**9.282 (<.001)**	n/a	**10.689 (<.001)**	n/a	**3.140 (.026)**	n/a
Aud	**4.552 (.004)**	n/a	**5.914 (.001)**	n/a	**17.183 (<.001)**	n/a	**9.574 (<.001)**	n/a
DMN	**4.167 (.007)**	n/a	**8.026 (<.001)**	n/a	2.526 (.058)	n/a	0.069 (.976)	n/a
FP	0.485 (.693)	n/a	1.009 (.389)	n/a	2.500 (.060)	n/a	0.864 (.460)	n/a
VAN	0.756 (.520)	n/a	0.385 (.764)	n/a	0.541 (.654)	n/a	1.070 (.362)	n/a
CO	**3.685 (.012)**	n/a	0.586 (.624)	n/a	**8.004 (<.001)**	n/a	**4.613 (.004)**	n/a
DAN	0.322 (.809)	n/a	0.377 (.770)	n/a	**2.959 (.033)**	n/a	1.060 (.366)	n/a
Sal	0.802 (.494)	n/a	1.026 (.381)	n/a	1.464 (.224)	n/a	1.095 (.351)	n/a
Between‐network								
Hand	1.485 (.219)	1.388 (.246)	1.849 (.138)	**7.927 (<.001)**	**5.588 (.001)**	n/a	n/a	n/a
Vis	0.482 (.695)	**4.154 (.007)**	0.104 (.958)	**12.094 (<.001)**	2.253 (.082)	n/a	n/a	n/a
Mouth	**3.238 (.023)**	1.545 (.203)	**3.012 (.030)**	1.758 (.155)	**6.757 (<.001)**	n/a	n/a	n/a
Aud	**4.872 (.003)**	0.757 (.519)	2.534 (.057)	1.898 (.130)	**13.543 (<.001)**	n/a	n/a	n/a
DMN	**3.953 (.009)**	**3.281 (.021)**	2.576 (.054)	1.350 (.258)	**7.915 (<.001)**	n/a	n/a	n/a
FP	0.117 (.950)	**4.293 (.006)**	2.311 (.076)	**6.737 (<.001)**	2.622 (.051)	n/a	n/a	n/a
VAN	**3.418 (.018)**	**4.682 (.003)**	1.203 (.309)	2.413 (.067)	**7.521 (<.001)**	n/a	n/a	n/a
CO	2.560 (.055)	1.138 (.334)	1.130 (.337)	1.879 (.133)	**12.345 (<.001)**	n/a	n/a	n/a
DAN	2.085 (.102)	1.352 (.258)	0.197 (.898)	**6.879 (<.001)**	1.999 (.114)	n/a	n/a	n/a
Sal	2.383 (.069)	2.167 (.092)	0.210 (.890)	**3.177 (.024)**	**9.599 (<.001)**	n/a	n/a	n/a

Note

Values represent *F*‐values (*p*‐values in parentheses) for each interaction and main effect; significant values are bolded.

**FIGURE 2:**
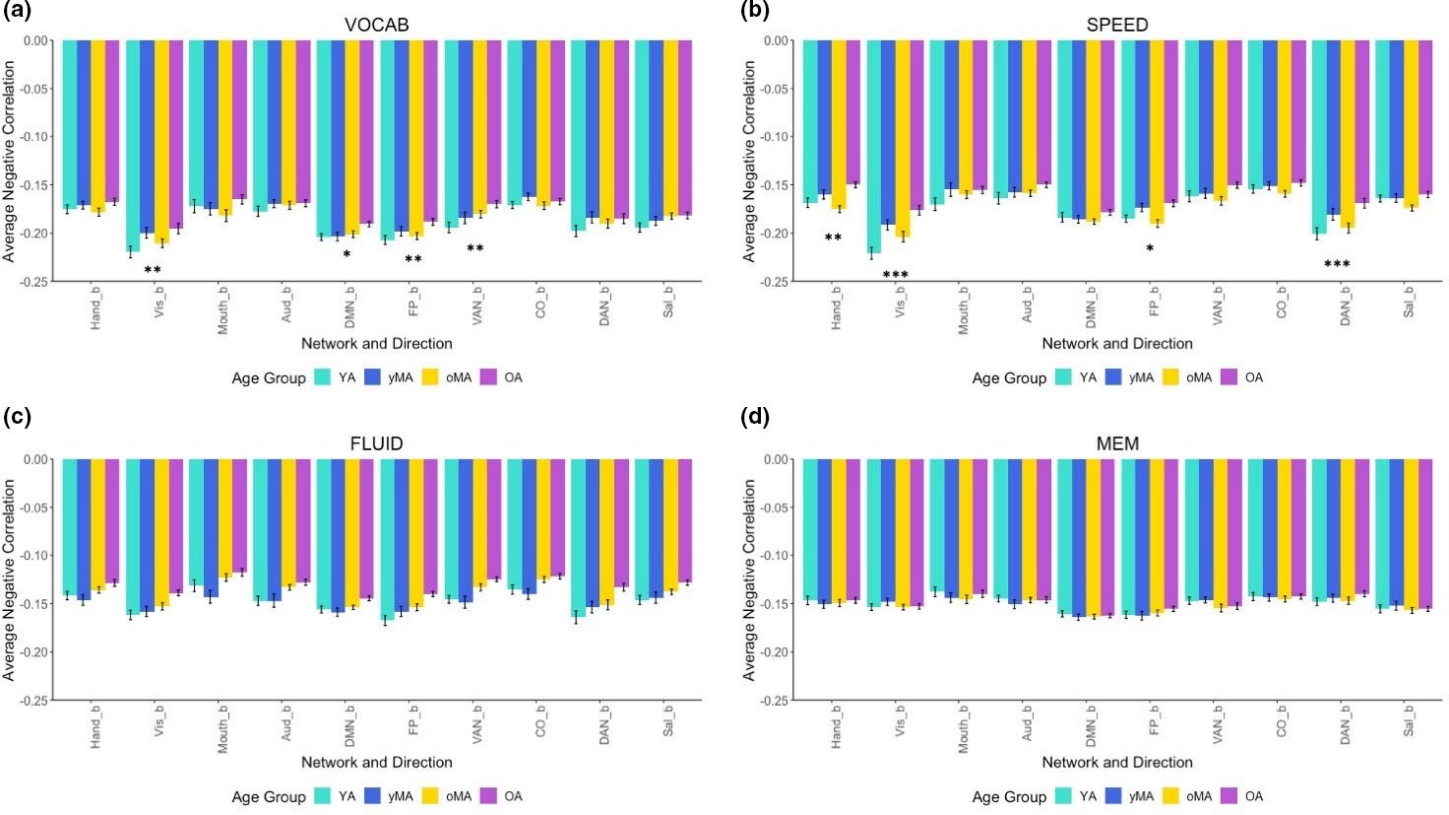
Average between‐network negative correlation for each age group, depicted for each task domain. Asterisks reflect significance of the difference between younger (YA) and older (OA) adults at each network (*p < .05, **p < .01, ***p < .001; all Bonferroni corrected within each domain). Networks are presented using acronyms defined in the text: Aud, Auditory; CO = Cingulo‐Opercular; DAN, Dorsal Attention Network; DMN, Default Mode Network; FP, Frontoparietal; Hand, Somatomotor Hand; Mouth, Somatomotor Mouth; Sal, Salience; VAN, Ventral Attention Network; Vis, Visual

A 4 (Domain: VOCAB, SPEED, FLUID, MEM) × 10 (Network) × 4 (Age Group: YA, yMA, oMA, OA) revealed a significant main effect of age group (*F*_3,194_ = 7.009, *p *< .001) on negative between‐network correlation strength. Additionally, there were significant interactions among domain and age group (*F*_9,882_ = 2.758, *p* = .003), network and age group (*F*_27,2,646_ = 1.732, *p* = .011), and domain and network and age group (*F*_81,7,938_ = 1.784, *p *< .001). The main effect of age suggested that, overall, older age was associated with weaker negative correlation strength (see Table 2, Tables S1–S4). Follow‐up analyses of the 3‐way interaction showed that there was an interaction between age and network on negative correlation strength during VOCAB (*F*_27,2,673_ = 1.593, *p* = .027) and SPEED (*F*_27,2,673_ = 3.377, *p *< .001) tasks, but not during FLUID (*F*_27,2,673_ = 1.295, *p* = .141) and MEM (*F*_27,2,673_ = 0.810, *p* = .743) tasks. Further, the main effect of age on negative correlation strength was significant for every domain except for MEM (VOCAB: *F*_3,297_ = 3.082, *p*‐.028; SPEED: *F*_3,297_ = 6.080, *p *< .001; FLUID: *F*_3,297_ = 6.982, *p *< .001; MEM: *F*_3,297_ = 0.441, *p* = .724). The interaction between age and network during VOCAB and SPEED showed that some between‐network negative correlations showed weakening with age across both tasks (i.e., Vis and FP networks), but some were specific to one task or the other (i.e., Hand and DAN between‐network negative correlations only showed an effect of age during SPEED tasks; see Figure 2, Table 2, Tables S1–S4).

### 3.2 System segregation

A 4 (Domain: VOCAB, SPEED, FLUID, MEM) × 2 (Network: Somatomotor versus. Association) × 4 (Age Group: YA, yMA, oMA, OA) MANCOVA revealed a significant main effect of age group (*F*_3,294_ = 6.528, *p *< .001) on system segregation. The interactions among network and age group (*F*_3,294_ = 1.695, *p* = .168), domain and age group (*F*_9,882_ = 0.839, *p* = .580), and network and domain and age group (*F*_9,882_ = 1.765, *p* = .071) were not significant (for other significant results, see Figure S1). The main effect of age group showed that younger adults (*m* = 0.118, *SD* = 0.185) showed greater system segregation than older adults (*m* = −0.013, *SD* = 0.184; mean difference = 0.131, *p *< .001; no other comparisons significant).

### 3.3 Graph theory metrics of global connectivity

A 4 (Domain: VOCAB, SPEED, FLUID, MEM) × 9 (Threshold: 2%–10%) × 4 (Age Group: YA, yMA, oMA, OA) MANCOVA revealed a significant interaction between domain and age group (*F*_9,882_ = 2.064, *p* = .030). The main effect of age group (*F*_3,294_ = 2.115, *p* = .098), and the interactions among threshold and age group (*F*_24,2,352_ = 0.477, *p* = .985) and domain and threshold and age group (*F*_72,7,056_ = 0.901, *p* = .710) were not significant. The interaction between age and domain was driven by YAs showing higher levels of global efficiency than oMAs (mean difference = 0.006, *p* = .015) and OAs (mean difference = 0.007, *p* = .003) during FLUID tasks (*F*_3,297_ = 4.718, *p* = .003), but no effect on age on global efficiency during VOCAB (*F*_3,297_ = 0.731, *p* = .534), SPEED (*F*_3,297_ = 1.682, *p* = .171), or MEM (*F*_3,297_ = 1.336, *p* = .263) tasks (see Figure S2).

A 4 (Domain: VOCAB, SPEED, FLUID, MEM) × 9 (Threshold: 2%–10%) × 4 (Age Group: YA, yMA, oMA, OA) MANCOVA revealed a significant main effect of age group (*F*_3,294_ = 8.927, *p *< .001) on modularity. Additionally, there were significant interactions among domain and age group (*F*_9,882_ = 2.791, *p* = .003), and threshold and age group (*F*_24,2,352_ = 2.896, *p *< .001). The interaction among domain, threshold, and age group was not significant (*F*_72,7,056_ = 0.578, *p* = .998). The main effect of age showed that YAs (mean difference = 0.019, *p *< .001) and yMAs (mean difference = 0.025, *p* = .004) had greater modularity than OAs, and YAs had greater modularity than oMAs (mean difference = 0.019, *p* = .047). The interaction between age and domain showed that there was a significant effect of age on modularity only during SPEED and FLUID tasks. Finally, the interaction between age group and threshold showed that the effect of age group on modularity differed by threshold (see Figure S3).

**FIGURE 3:**
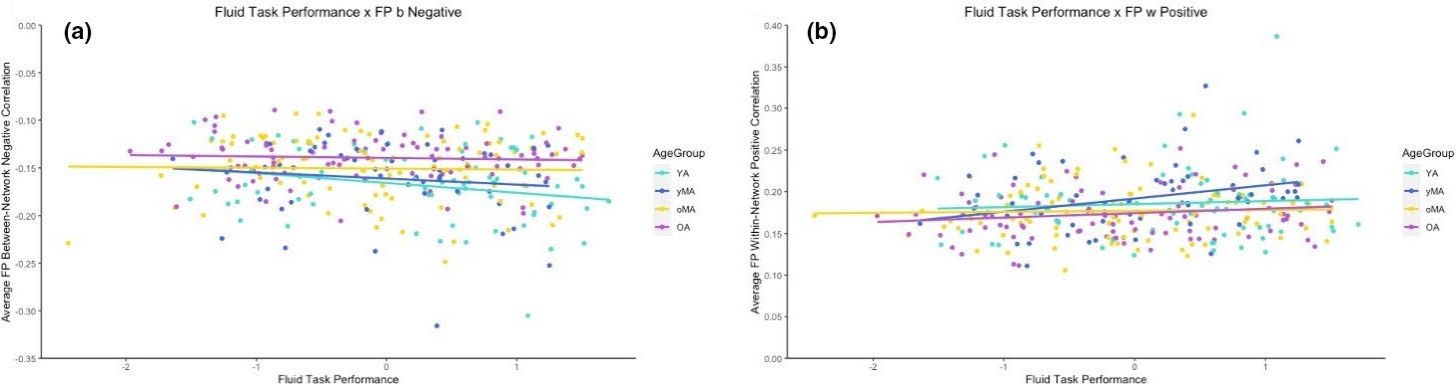
Visualization of correlations between FLUID task performance and average Frontoparietal (FP) between‐network connectivity in both the negative (a) and positive (b) directions. The correlation with negative FP between‐network connectivity represents a correlation that become nonsignificant after controlling for age, while the correlation with positive FP between‐network connectivity represents a correlation that remained significant after controlling for age

#### 3.3.1 Correlational brain–behavior analyses

Results from the exploratory correlational analyses showed generally that whole‐brain graph theory metrics show few relationships with task performance, and however, whole‐brain system segregation was related to MEM task performance (see Table 3; *p* = .002). Further, FLUID and MEM performance was also related to several network‐based average correlation metrics, while VOCAB and SPEED performance showed fewer associations with these network‐based metrics (see Table 3). Further, while many of these relationships persisted after accounting for participant age, several in the FLUID domain became nonsignificant (*p* > .05) after controlling for this effect.

**TABLE 3:** Pearson partial correlation coefficients for relationships between connectivity metrics and task domain z‐scores (controlling for years of education and gender)

Connectivity metric		VOCAB	SPEED	FLUID	MEM
System segregation	Association	−0.070 (.227)	−0.007 (.900)	−0.010 (.865)	**0.117 (.042)**
	Somatomotor	−0.059 (.306)	−0.042 (.464)	0.026 (.649)	**0.137 (.018)**
	Whole‐Brain	−0.065 (.261)	−0.066 (.256)	0.006 (.918)	0.180 (.002)*
Hand	w pos	−0.068 (.239)	−0.132 (.023)*	0.044 (.450)	0.013 (.823)
	b pos	−0.041 (.484)	0.041 (.483)	0.154 (.007)*	−0.018 (.751)
	b neg	0.026 (.655)	0.068 (.244)	**−0.125 (.030)**	−0.009 (.882)
Vis	w pos	−0.082 (.158)	0.038 (.514)	0.108 (.063)	0.052 (.369)
	b pos	−0.093 (.109)	0.000 (.997)	**0.114 (.049)**	**−0.138 (.017)**
	b neg	0.073 (.210)	0.108 (.062)	**−0.119 (.039)**	0.006 (.918)
Mouth	w pos	−0.056 (.330)	0.005 (.926)	**0.122 (.035)**	0.007 (.903)
	b pos	−0.093 (.109)	0.005 (.929)	0.089 (.124)	**−0.118 (.041)**
	b neg	0.094 (.105)	0.087 (.133)	−0.042 (.472)	0.074 (.200)
Aud	w pos	−0.077 (.182)	−0.098 (.090)	0.107 (.064)	−0.009 (.875)
	b pos	**−0.130 (.024)**	−0.098 (.090)	0.092 (.113)	**−0.122 (.035)**
	b neg	0.006 (.918)	0.064 (.269)	−0.076 (.188)	−0.013 (.829)
DMN	w pos	−0.069 (.236)	−0.060 (.299)	0.065 (.258)	−0.043 (.463)
	b pos	−0.095 (.099)	−0.105 (.069)	0.109 (.060)	−0.113 (.051)*
	b neg	0.084 (.146)	0.077 (.186)	**−0.137 (.018)**	0.071 (.222)
FP	w pos	0.060 (.303)	−0.023 (.692)	0.093 (.107)	−0.035 (.541)
	b pos	−0.060 (.302)	−0.048 (.408)	0.182 (.002)*	−0.070 (.224)
	b neg	0.068 (.242)	0.068 (.243)	**−0.142 (.014)**	0.047 (.415)
VAN	w pos	−0.108 (.063)	0.128 (.026)*	0.078 (.178)	−0.009 (.877)
	b pos	−0.176 (.002)*	−0.069 (.233)	0.110 (.057)	−0.140 (.015)*
	b neg	0.095 (.102)	0.044 (.444)	−0.095 (.102)	0.076 (.188)
CO	w pos	−0.067 (.248)	−0.104 (.072)	0.084 (.149)	0.021 (.714)
	b pos	−0.061 (.289)	−0.081 (.160)	**0.125 (.030)**	−0.065 (.263)
	b neg	0.072 (.216)	0.016 (.776)	−0.111 (.054)	0.014 (.809)
DAN	w pos	−0.025 (.662)	−0.084 (.147)	0.223 (<.001)*	0.066 (.254)
	b pos	−0.079 (.171)	−0.034 (.554)	0.160 (.005)*	−0.030 (.600)
	b neg	0.083 (.150)	0.080 (.168)	−0.071 (.219)	−0.033 (.572)
Sal	w pos	0.009 (.880)	−0.164 (.004)*	0.053 (.364)	−0.040 (.486)
	b pos	−0.020 (.734)	−0.051 (.381)	**0.146 (.012)**	0.012 (.841)
	b neg	0.099 (.088)	0.045 (.441)	−0.145 (.012)*	**0.133 (.021)**
GE	2%	−0.026 (.657)	**0.116 (.044)**	−0.135 (.020)*	−0.014 (.809)
	3%	−0.016 (.778)	0.112 (.053)	−0.101 (.082)	−0.026 (.654)
	4%	−0.018 (.759)	**0.118 (.041)**	−0.097 (.094)	−0.027 (.643)
	5%	−0.030 (.603)	0.120 (.038)*	−0.035 (.544)	−0.035 (.550)
	6%	−0.032 (.583)	0.108 (.061)	0.001 (.986)	−0.048 (.406)
	7%	−0.074 (.200)	0.122 (.035)*	0.052 (.372)	−0.067 (.250)
	8%	−0.098 (.09)*	0.123 (.033)*	0.088 (.129)	−0.085 (.141)
	9%	−0.110 (.056)*	0.109 (.058)	0.106 (.067)	−0.101 (.080)
	10%	−0.126 (.030)*	0.104 (.071)	**0.128 (.027)**	−0.105 (.068)
Mod	2%	−0.034 (.562)	0.037 (.523)	−0.068 (.244)	0.008 (.896)
	3%	−0.031 (.592)	−0.004 (.951)	−0.046 (.430)	0.030 (.601)
	4%	−0.047 (.415)	−0.021 (.721)	−0.025 (.662)	0.016 (.783)
	5%	−0.041 (.477)	−0.040 (.494)	0.009 (.870)	0.029 (.612)
	6%	−0.036 (.535)	−0.050 (.387)	0.034 (.561)	0.008 (.892)
	7%	−0.036 (.530)	−0.050 (.386)	0.052 (.365)	−0.004 (.949)
	8%	−0.030 (.609)	−0.052 (.372)	0.062 (.285)	−0.005 (.929)
	9%	−0.044 (.448)	−0.062 (.284)	0.076 (.189)	−0.001 (.987)
	10%	−0.038 (.516)	−0.075 (.196)	0.099 (.088)	−0.003 (.960)

Note

Values represent raw Pearson partial correlation coefficients (*p*‐values in parentheses) for each pairing; coefficients with *p*‐values < .05 are bolded. Coefficients with *p*‐values < .05 after additionally controlling for participant age are followed by an asterisk.

Abbreviations: b neg, average between‐network negative correlation; b pos, average between‐network positive correlation; GE, global efficiency; Mod, modularity; SS, system segregation; w pos, average within‐network positive correlation.

## 4 DISCUSSION

Results from the present study show that age is associated with differences in a variety of measures of functional connectivity across several cognitive task domains. Further, interactions between age and task domain indicate that in‐scanner task domain affects whether and how age effects are discovered. For positive correlations, this interaction shows that the auditory network shows an effect of age across all tasks ; however, cognitive networks may or may not show an effect of age depending upon the task being performed. For negative correlations, effects of age were only observed during VOCAB and SPEED tasks, and the networks affected differed between these two tasks. When examining graph theory measures, global efficiency and modularity also showed interactions between age and task, such that age had an effect on global efficiency only during FLUID tasks, and age had an effect on modularity during both SPEED and FLUID tasks.

While studies conducted at rest, or during just one cognitive task, show specific effects of age on functional connectivity metrics, the results presented here suggest that the effect of age may not be omnipresent and may be more or less apparent depending upon the choice of cognitive task. For example, age showed very little effect on functional connectivity during memory tasks (only on the average positive correlation in a few networks); however, the effects of age seem to be quite robust and readily observed during FLUID tasks. These results cannot simply be explained by presence or lack of behavioral differences on these tasks, since older adults performed significantly worse than younger adults on MEM tasks and marginally worse on FLUID tasks (see Table 1). This not only suggests that older adults are not unilaterally impaired (relative to younger adults) on metrics of functional connectivity, but also suggests that task choice can have a significant effect on results of functional connectivity analysis. Thus, these results provide strong evidence that task choice plays a significant role in studies assessing the effect of participant age on functional connectivity and therefore must play a critical role in both experimental design and in interpretation of results from studies examining the role of aging on functional connectivity computed during a task.

Findings from the present study mirror some findings from past studies investigating the effect of age on functional connectivity at rest (Betzel et al., 2014; Chan et al., 2014; Geerligs, Renken, et al., 2015; Iordan et al., 2017), showing that aging is associated with a reduction in within‐network connectivity, and a generally less modular/segregated brain. However, results from the present study showed that the cognitive task being performed affected the presence and extent of these effects. Further, unlike results from several of these previous studies showing an age‐related increase in between‐network connectivity, any differences in between‐network connectivity as a function of participant age revealed a weakening of between‐network connections with age.

In a recent study, our group used a nearly identical set of connectivity measures to characterize the effect of aging on functional connectivity during a resting state scan (Varangis, Habeck, et al., 2019). By using the same processing stream and analyzing many of these same metrics during 11 cognitive tasks, the present study allows for direct qualitative comparisons between patterns of results obtained during a resting state scan with those obtained during performance of cognitive tasks reflecting 4 overarching cognitive domains. Results from the present study largely mirror many of these findings at rest—that, generally, older age is associated with weaker within‐network positive correlations in several networks and reduced system segregation across the whole brain. However, results from analysis of the task‐based data show a more consistent effect of age on within‐ and between‐network correlations during performance of fluid reasoning tasks, and fewer effects of age on connectivity values during processing speed and memory tasks. Further, results from the present study also showed a weakening effect of age on average between‐network negative correlations during vocabulary and processing speed tasks, an effect of age on global efficiency during performance of fluid reasoning tasks, and an effect of age on modularity during performance of processing speed and fluid reasoning tasks. Thus, results from the present task‐based connectivity analyses were largely consistent with these results obtained during a resting state scan; however, they showed that performance of a cognitive task may result in exaggerated (fluid reasoning tasks) or dampened (memory tasks) effects of age on functional connectivity metrics. As such, studies specifically examining connectivity during rest, or during one specific cognitive task, must consider how the type of scan may have affected the patterns of results being observed.

Results from this study also corroborate and extend previous findings in this sample showing that specific connections among pairs of cognitive networks (modeled as latent network factors) differ as a function of cognitive task (Varangis, Razlighi, et al., 2019). The present study builds on these findings by utilizing more standard metrics of functional connectivity computed identically for each participant, incorporating whole‐brain metrics of connectivity, examining within‐network connectivity for network‐based metrics, and by including somatomotor networks in the analyses. More specifically, results from this previous study showed that older age is associated with differences in connections between several network pairs and that performance of a task is associated with altered patterns of connectivity between 6 cognitive networks, but that there was no interaction between participant age and task domain in predicting the strength of latent between‐network connectivity. In the present study, network connections were modeled in a more simplistic way, reflecting the average correlation within each network, and the average correlation from one network to all other networks. Results from the present study, therefore, are not directly comparable with these results, but suggest that age does interact with task domain in affecting the strength of correlations within and between networks. That being said, the present set of results showed that older age tended to generally be associated with weaker correlations within and between networks, while data from the previous study found certain network connections that were stronger in older age (i.e., FP‐Sal, FP‐Memory Network), and some that were weaker in older age (i.e., CO‐Sal, Memory Network‐DMN, and Sal‐DMN). While this may seem contradictory to the present results, this difference could reflect the granularity of between‐network correlation analysis (averaged over all other networks vs. modeled between pairs of networks), or the complexity of the latent factor modeling in the earlier paper. Additionally, these effects were observed across all tasks and were not probed at the task level due to a nonsignificant interaction among age group, task domain, and connection (network pairing). Thus, results from the present study complement and crucially build upon results from this previous work and provide evidence that task domain interacts with participant age in affecting a variety of more standard metrics of functional connectivity.

The present results also extend those of previous studies finding differences in the effect of age across multiple tasks (Archer et al., 2016; Burianova et al., 2015; Geerligs, Rubinov, et al., 2015). Two of these studies consistently found that performance of a task altered functional connectivity patterns in both younger and older adults, and importantly, found that age‐related differences in functional connectivity may be more readily observed during a task (Archer et al., 2016; Geerligs, Rubinov, et al., 2015). The third study, rather than comparing task conditions to a resting scan, compared functional connectivity across 2 levels of an n‐back task (Burianova et al., 2015). The authors of this study found that older adults showed less modulation of functional connectivity as a function of task load, suggesting that older adults are not as able to flexibly alter connectivity in response to changes in task demands. One aspect of these studies to note is that while they all looked at functional connectivity during multiple task conditions, the tasks were primarily targeting executive function (Archer et al., 2016; Burianova et al., 2015) or sensorimotor processing (Geerligs, Rubinov, et al., 2015). Critically, the studies did not compare functional connectivity across multiple cognitive domains, as in the present study. Thus, while these results generally support the results of the current study that show age‐related differences in functional connectivity during task performance, they cannot speak to the domain‐related differences, or interactions between age and domain, also observed in the results presented here.

Finally, given the scope of analyses conducted in the present study, brain–behavior correlational relationships were largely exploratory and thus interpreted with caution. That being said, several interesting patterns emerged: (a) System segregation and average network‐based correlation metrics seemed to show more of a relationship with performance than the whole‐brain graph theory metrics; (b) FLUID and MEM tasks seemed to evoke more potential associations between functional connectivity and task performance; and (c) positive correlations within and between networks tended to be positively correlated with task performance, while negative correlations between networks tended to be negatively correlated with task performance (with the exception of the salience network and MEM task performance). This somewhat mirrors previous studies finding that more negatively correlated activity between the DMN and cognitive networks was associated with better task performance. While the present study did not specifically interrogate connectivity between pairs of networks, the findings generally suggest that stronger negative correlations between networks may be associated with better task performance, but that stronger positive correlations between networks may also be associated with better task performance. This suggests that networks may adaptively engage in collaborative or antagonistic relationships with other networks in order to respond to the demands of the task. Importantly, our results suggest that these patterns of network interactions that either aid or detract from task performance may vary as a function of the task being conducted in the scanner. Further, while many of these relationships persisted after controlling for participant age, several correlations became nonsignificant. In visualizing some of these relationships that became nonsignificant after controlling for age, there was a trend toward differing relationships between cognition and connectivity as a function of age. For example, in examining the relationship between FP between‐network connectivity and FLUID task performance, stronger positive between‐network connectivity was associated with better performance on the task even after controlling for age, while negative between‐network connectivity was not (see Figure 3). When viewed as a function of age group, this pattern may suggest that older and younger adults show different relationships between negative FP between‐network connectivity and task performance, such that younger adults show a more negative relationship between this metric and performance, while older adults show no relationship between this metric and performance. While formal testing of these trends was beyond the scope of the present analyses, future studies should more specifically examine whether and how age may moderate the effect of task‐based functional connectivity on task performance.

The present study, however, is not without limitations. First, the present study did not compare functional connectivity metrics between task and rest. While this limits the ability to draw conclusions about the difference in age effects between resting state and various tasks, the study does find that task domain affects the presence and magnitude of age effects across every metric of functional connectivity included in the present study. This conclusion that task domain affects these metrics is key in determining the true effect of age on functional neural connectivity across multiple cognitive states. Second, the present study utilized predefined functional networks as defined by Power et al. (2011) and did not identify these functional networks based on their localization in this sample. While this might slightly weaken the applicability of these networks in this sample, the fact that the present study found differences based on age and task domain in these networks suggests that their definition may be relevant and useful to external samples.

Based on the results of the present study, future studies should continue to explore age‐related differences in functional connectivity across different cognitive tasks in order to assess domain specificity of age‐related differences in functional connectivity. Results from the present study suggest that fluid reasoning tasks may be more sensitive to age‐related differences in functional connectivity than episodic memory tasks. Since age‐related behavioral differences are readily observed in memory tasks and marginally evident in fluid reasoning tasks, it may be plausibly hypothesized that age‐related decrements in fluid reasoning task performance may arise alongside or as a function of age‐related differences in functional connectivity, however, since few differences in connectivity were observed during episodic memory tasks, a functional connectivity‐related biomarker for age‐related memory decline may be less clearly applicable. That being said, future studies should further delve into this effect by more thoroughly exploring the effect of age on functional connectivity during a variety of memory tasks across multiple modalities and components of memory.

### 4.1 Conclusions

Results from the present study suggest that a variety of measures of functional connectivity may be sensitive to age‐related differences during performance of cognitive tasks tapping into four domains: vocabulary, processing speed, fluid reasoning, and episodic memory. These differences seem to be more extensive during fluid reasoning and processing speed tasks and seem to show a general trend toward older adults showing reduced functional connectivity within and between predefined networks, reduced system segregation, and lower global efficiency and modularity.

## CONFLICT OF INTEREST

The authors declared no potential conflicts of interest with respect to the research, authorship, and/or publication of this article.

## AUTHOR CONTRIBUTION

YS and CH designed the parent study and oversaw the data collection. YS and CH consulted on analysis techniques and statistical methodology. EV analyzed the task‐based brain imaging and behavioral data, and wrote the original draft. All authors reviewed and edited the final manuscript.

### Peer Review

The peer review history for this article is available at https://publons.com/publon/10.1002/brb3.1954.

## Supporting information

Supplementary Material

## Data Availability

The datasets and code generated for this study are available on request to the corresponding author and are subject to acceptance of a formal data sharing agreement.

## References

[brb31954-bib-0001] Andrews‐Hanna, J. R. , Snyder, A. Z. , Vincent, J. L. , Lustig, C. , Head, D. , Raichle, M. E. , & Buckner, R. L. (2007). Disruption of large‐scale brain systems in advanced aging. Neuron, 56(5), 924–935. 10.1016/j.neuron.2007.10.038 18054866 PMC2709284

[brb31954-bib-0002] Archer, J. A. , Lee, A. , Qiu, A. , & Chen, S. H. (2016). A comprehensive analysis of connectivity and aging over the adult life span. Brain Connect, 6(2), 169–185. 10.1089/brain.2015.0345 26652914

[brb31954-bib-0003] Betzel, R. F. , Byrge, L. , He, Y. , Goni, J. , Zuo, X. N. , & Sporns, O. (2014). Changes in structural and functional connectivity among resting‐state networks across the human lifespan. NeuroImage, 102(Pt 2), 345–357. 10.1016/j.neuroimage.2014.07.067 25109530

[brb31954-bib-0101] Birn, R. M. , Diamond, J. B. , Smith, M. A. , & Bandettini, P. A. (2006). Separating respiratory‐variation‐related fluctuations from neuronal‐activity‐related fluctuations in fMRI. NeuroImage, 31(4), 1536–1548. 10.1016/j.neuroimage.2006.02.048 16632379

[brb31954-bib-0004] Burianova, H. , Marstaller, L. , Choupan, J. , Sepehrband, F. , Ziaei, M. , & Reutens, D. (2015). The relation of structural integrity and task‐related functional connectivity in the aging brain. Neurobiology of Aging, 36(10), 2830–2837. 10.1016/j.neurobiolaging.2015.07.006 26234754

[brb31954-bib-0005] Campbell, K. L. , Grady, C. L. , Ng, C. , & Hasher, L. (2012). Age differences in the frontoparietal cognitive control network: Implications for distractibility. Neuropsychologia, 50(9), 2212–2223. 10.1016/j.neuropsychologia.2012.05.025 22659108 PMC4898951

[brb31954-bib-0102] Carp, J. (2013). Optimizing the order of operations for movement scrubbing: Comment on Power et al NeuroImage, 76, 436–438. 10.1016/j.neuroimage.2011.12.061 22227884

[brb31954-bib-0103] Chan, M. Y. , Alhazmi, F. H. , Park, D. C. , Savalia, N. K. , & Wig, G. S. (2017). Resting‐state network topology differentiates task signals across the adult life span. Journal of Neuroscience, 37(10), 2734–2745. 10.1523/jneurosci.2406-16.2017 28174333 PMC5354325

[brb31954-bib-0006] Chan, M. Y. , Park, D. C. , Savalia, N. K. , Petersen, S. E. , & Wig, G. S. (2014). Decreased segregation of brain systems across the healthy adult lifespan. Proceedings of the National Academy of Sciences of the United States of America, 111(46), E4997–E5006. 10.1073/pnas.1415122111 25368199 PMC4246293

[brb31954-bib-0104] Dale, A. M. , Fischl, B. , & Sereno, M. I. (1999). Cortical surface‐based analysis. I. Segmentation and surface reconstruction. NeuroImage, 9(2), 179–194. 10.1006/nimg.1998.0395 9931268

[brb31954-bib-0105] Fischl, B. , Salat, D. H. , Busa, E. , Albert, M. , Dieterich, M. , Haselgrove, C. , van der Kouwe, A. , Killiany, R. , Kennedy, D. , Klaveness, S. , Montillo, A. , Makris, N. , Rosen, B. , & Dale, A. M. (2002). Whole brain segmentation: automated labeling of neuroanatomical structures in the human brain. Neuron, 33(3), 341–355. 11832223 10.1016/s0896-6273(02)00569-x

[brb31954-bib-0106] Fischl, B. , van der Kouwe, A. , Destrieux, C. , Halgren, E. , Segonne, F. , Salat, D. H. , Busa, E. , Seidman, L. J. , Goldstein, J. , Kennedy, D. , Caviness, V. , Makris, N. , Rosen, B. , & Dale, A. M. (2004). Automatically parcellating the human cerebral cortex. Cerebral Cortex, 14(1), 11–22. 14654453 10.1093/cercor/bhg087

[brb31954-bib-0007] Geerligs, L. , Maurits, N. M. , Renken, R. J. , & Lorist, M. M. (2014). Reduced specificity of functional connectivity in the aging brain during task performance. Human Brain Mapping, 35(1), 319–330. 10.1002/hbm.22175 22915491 PMC6869200

[brb31954-bib-0008] Geerligs, L. , Renken, R. J. , Saliasi, E. , Maurits, N. M. , & Lorist, M. M. (2015). A brain‐wide study of age‐related changes in functional connectivity. Cerebral Cortex, 25(7), 1987–1999. 10.1093/cercor/bhu012 24532319

[brb31954-bib-0009] Geerligs, L. , Rubinov, M. , Cam, C. A. N. , & Henson, R. N. (2015). State and Trait Components Of Functional Connectivity: Individual differences vary with mental state. Journal of Neuroscience, 35(41), 13949–13961. 10.1523/jneurosci.1324-15.2015 26468196 PMC4604231

[brb31954-bib-0010] Iordan, A. D. , Cooke, K. A. , Moored, K. D. , Katz, B. , Buschkuehl, M. , Jaeggi, S. M. , Jonides, J. , Peltier, S. J. , Polk, T. A. , & Reuter‐Lorenz, P. A. (2017). Aging and network properties: Stability over time and links with learning during working memory training. Frontiers in Aging Neuroscience, 9, 419 10.3389/fnagi.2017.00419 29354048 PMC5758500

[brb31954-bib-0107] Jenkinson, M. , Bannister, P. , Brady, M. , & Smith, S. (2002). Improved optimization for the robust and accurate linear registration and motion correction of brain images. NeuroImage, 17(2), 825–841. 12377157 10.1016/s1053-8119(02)91132-8

[brb31954-bib-0108] Jenkinson, M. , Beckmann, C. F. , Behrens, T. E. , Woolrich, M. W. , & Smith, S. M. (2012). FSL. NeuroImage, 62(2), 782–790. 10.1016/j.neuroimage.2011.09.015 21979382

[brb31954-bib-0109] Jenkinson, M. , & Smith, S. (2001). A global optimisation method for robust affine registration of brain images. Medical Image Analysis, 5(2), 143–156. 11516708 10.1016/s1361-8415(01)00036-6

[brb31954-bib-0011] King, B. R. , van Ruitenbeek, P. , Leunissen, I. , Cuypers, K. , Heise, K.‐F. , Santos Monteiro, T. , Hermans, L. , Levin, O. , Albouy, G. , Mantini, D. , & Swinnen, S. P. (2017). Age‐related declines in motor performance are associated with decreased segregation of large‐scale resting state brain networks. Cerebral Cortex, 28(12), 4390–4402. 10.1093/cercor/bhx297 PMC6215458 29136114

[brb31954-bib-0110] Latora, V. , & Marchiori, M. (2001). Efficient behavior of small‐world networks. Physical Review Letters, 87(19), 198701 10.1103/PhysRevLett.87.198701 11690461

[brb31954-bib-0012] Miller, S. L. , Celone, K. , DePeau, K. , Diamond, E. , Dickerson, B. C. , Rentz, D. , Pihlajamaki, M. , & Sperling, R. A. (2008). Age‐related memory impairment associated with loss of parietal deactivation but preserved hippocampal activation. Proceedings of the National Academy of Sciences of the United States of America, 105(6), 2181–2186. 10.1073/pnas.0706818105 18238903 PMC2538895

[brb31954-bib-0111] Newman, M. E. (2006). Modularity and community structure in networks. Proceedings of the National Academy of Sciences of the United States of America, 103(23), 8577–8582. 10.1073/pnas.0601602103 16723398 PMC1482622

[brb31954-bib-0013] Onoda, K. , Ishihara, M. , & Yamaguchi, S. (2012). Decreased functional connectivity by aging is associated with cognitive decline. Journal of Cognitive Neuroscience, 24(11), 2186–2198. 10.1162/jocn_a_00269 22784277

[brb31954-bib-0014] Parkes, L. , Fulcher, B. , Yucel, M. , & Fornito, A. (2018). An evaluation of the efficacy, reliability, and sensitivity of motion correction strategies for resting‐state functional MRI. NeuroImage, 171, 415–436. 10.1016/j.neuroimage.2017.12.073 29278773

[brb31954-bib-0015] Power, J. D. , Barnes, K. A. , Snyder, A. Z. , Schlaggar, B. L. , & Petersen, S. E. (2012). Spurious but systematic correlations in functional connectivity MRI networks arise from subject motion. NeuroImage, 59(3), 2142–2154. 10.1016/j.neuroimage.2011.10.018 22019881 PMC3254728

[brb31954-bib-0112] Power, J. D. , Cohen, A. L. , Nelson, S. M. , Wig, G. S. , Barnes, K. A. , Church, J. A. , Vogel, A. C. , Laumann, T. O. , Miezin, F. M. , Schlaggar, B. L. , & Petersen, S. E. (2011). Functional network organization of the human brain. Neuron, 72(4), 665–678. 10.1016/j.neuron.2011.09.006 22099467 PMC3222858

[brb31954-bib-0016] Prakash, R. S. , Heo, S. , Voss, M. W. , Patterson, B. , & Kramer, A. F. (2012). Age‐related differences in cortical recruitment and suppression: Implications for cognitive performance. Behavioral Brain Research, 230(1), 192–200. 10.1016/j.bbr.2012.01.058 22348896

[brb31954-bib-0017] Razlighi, Q. R. , Habeck, C. , Barulli, D. , & Stern, Y. (2017). Cognitive neuroscience neuroimaging repository for the adult lifespan. NeuroImage, 144(Pt B), 294–298. 10.1016/j.neuroimage.2015.08.037 26311605 PMC4766063

[brb31954-bib-0113] Razlighi, Q. R. , Habeck, C. , Steffener, J. , Gazes, Y. , Zahodne, L. B. , MacKay‐Brandt, A. , & Stern, Y. (2014). Unilateral disruptions in the default network with aging in native space. Brain and Behavior, 4(2), 143–157. 10.1002/brb3.202 24683508 PMC3967531

[brb31954-bib-0114] Rubinov, M. , & Sporns, O. (2010). Complex network measures of brain connectivity: Uses and interpretations. NeuroImage, 52(3), 1059–1069. 10.1016/j.neuroimage.2009.10.003 19819337

[brb31954-bib-0018] Sala‐Llonch, R. , Junqué, C. , Arenaza‐Urquijo, E. M. , Vidal‐Piñeiro, D. , Valls‐Pedret, C. , Palacios, E. M. , Domènech, S. , Salvà, A. , Bargalló, N. , & Bartrés‐Faz, D. (2014). Changes in whole‐brain functional networks and memory performance in aging. Neurobiology of Aging, 35(10), 2193–2202. 10.1016/j.neurobiolaging.2014.04.007 24814675

[brb31954-bib-0019] Sala‐Llonch, R. , Pena‐Gomez, C. , Arenaza‐Urquijo, E. M. , Vidal‐Pineiro, D. , Bargallo, N. , Junque, C. , & Bartres‐Faz, D. (2012). Brain connectivity during resting state and subsequent working memory task predicts behavioural performance. Cortex, 48(9), 1187–1196. 10.1016/j.cortex.2011.07.006 21872853

[brb31954-bib-0020] Salthouse, T. A. (2009). Decomposing age correlations on neuropsychological and cognitive variables. Journal of the International Neuropsychological Society, 15(5), 650–661. 10.1017/s1355617709990385 19570312 PMC3633567

[brb31954-bib-0021] Salthouse, T. A. , & Ferrer‐Caja, E. (2003). What needs to be explained to account for age‐related effects on multiple cognitive variables? Psychology and Aging, 18(1), 91–110. 10.1037/0882-7974.18.1.91 12641315

[brb31954-bib-0022] Song, J. , Birn, R. M. , Boly, M. , Meier, T. B. , Nair, V. A. , Meyerand, M. E. , & Prabhakaran, V. (2014). Age‐related reorganizational changes in modularity and functional connectivity of human brain networks. Brain Connect, 4(9), 662–676. 10.1089/brain.2014.0286 25183440 PMC4238253

[brb31954-bib-0023] Spreng, R. N. , Stevens, W. D. , Viviano, J. D. , & Schacter, D. L. (2016). Attenuated anticorrelation between the default and dorsal attention networks with aging: Evidence from task and rest. Neurobiology of Aging, 45, 149–160. 10.1016/j.neurobiolaging.2016.05.020 27459935 PMC5003045

[brb31954-bib-0024] Stern, Y. , Habeck, C. , Steffener, J. , Barulli, D. , Gazes, Y. , Razlighi, Q. , Shaked, D. , & Salthouse, T. (2014). The Reference Ability Neural Network Study: Motivation, design, and initial feasibility analyses. NeuroImage, 103, 139–151. 10.1016/j.neuroimage.2014.09.029 25245813 PMC4312259

[brb31954-bib-0025] Varangis, E. , Habeck, C. G. , Razlighi, Q. R. , & Stern, Y. (2019). The effect of aging on resting state connectivity of predefined networks in the brain. Frontiers in Aging Neuroscience, 11, 234 10.3389/fnagi.2019.00234 31555124 PMC6737010

[brb31954-bib-0026] Varangis, E. , Razlighi, Q. , Habeck, C. , Fisher, Z. , & Stern, Y. (2019). Between‐network functional connectivity is modified by age and cognitive task domain. Journal of Cognitive Neuroscience, 34(4), 10.1162/jocn_a_01368 PMC6414048 30605005

[brb31954-bib-0027] Wang, L. , LaViolette, P. , O'Keefe, K. , Putcha, D. , Bakkour, A. , Van Dijk, K. R. A. , Pihlajamäki, M. , Dickerson, B. C. , & Sperling, R. A. (2010). Intrinsic connectivity between the hippocampus and posteromedial cortex predicts memory performance in cognitively intact older individuals. NeuroImage, 51(2), 910–917. 10.1016/j.neuroimage.2010.02.046 20188183 PMC2856812

[brb31954-bib-0115] Wig, G. S. (2017). Segregated systems of human brain networks. Trends in Cognitive Sciences, 21(12), 981–996. 10.1016/j.tics.2017.09.006 29100737

[brb31954-bib-0028] Zonneveld, H. I. , Pruim, R. H. R. , Bos, D. , Vrooman, H. A. , Muetzel, R. L. , Hofman, A. , Rombouts, S. A. R. B. , van der Lugt, A. , Niessen, W. J. , Ikram, M. A. , & Vernooij, M. W. (2019). Patterns of functional connectivity in an aging population: The Rotterdam Study. NeuroImage, 189, 432–444. 10.1016/j.neuroimage.2019.01.041 30659958

