# Supplementary material for: Task‐based functional connectivity in aging: How task and connectivity methodology affect discovery of age effects
Source: Brain Behav. 2020 Nov 18;11(1):e01954. doi: 10.1002/brb3.1954 (PMC7821554; doi:10.1002/brb3.1954)

**Supporting Information for “Task-Based Functional Connectivity in Aging: How Task and Connectivity Methodology Affect Discovery of Age Effects”**

Full Results text for all MANCOVA analyses:

**3.1 Positive/Negative Correlation Weights.** A 4 (Domain: VOCAB, SPEED, FLUID, MEM) x 10 (Network) x 2 (Correlation direction: within, between) x 4 (Age Group: YA, yMA, oMA, OA) revealed significant main effects of domain (F_3,822_=47.119, p<.001), network (F_9,2466_=127.455, p<.001), direction (F_1,274_=6.674, p=0.010), and age group (F_3,274_=11.551, p<.001) on positive correlation strength. Further, there were significant interactions among domain and age group (F_9,822_=2.185, p=0.021), network and age group (F_27,2466_=2.552, p<.001), direction and age group (F_3,274_=13.859, p<.001), domain and network (F_27,7398_=12.828, p<.001), domain and direction (F_3,822_=34.263, p<.001), network and direction (F_9,2466_=76.809, p<.001), domain and network and age group (F_81,7398_=1.503, p=0.002), network and direction and age group (F_27,2466_=2.670, p<.001), domain and network and direction (F_27,7398_=7.215, p<.001), and domain and network and direction and age group (F_81,7398_=1.575, p=0.001). The interaction among domain, direction, and age group was not significant (F_9,822_=0.386, p=0.942). Follow-up analyses probing the 4-way interaction showed that age had a dampening effect on positive correlation strength for differing networks depending upon the domain being analyzed (see Figure 1, Table 2, Supplementary Tables S1-3). The visual, somatomotor mouth, and auditory networks showed an effect of age on within-network correlations across all four domains, however other networks only showed an effect of age on within- or between-network correlation strength in specific domains (i.e., Hand, CO, and Sal between-network correlation strength only showed an effect of age during FLUID tasks).

A 4 (Domain: VOCAB, SPEED, FLUID, MEM) x 10 (Network) x 4 (Age Group: YA, yMA, oMA, OA) revealed significant main effects of domain (F_3,882_=119.454, p<.001), network (F_9,2646_=80.626, p<.001), and age group (F_3,194_=7.009, p<.001) on negative between-network correlation strength. Additionally, there were significant interactions among domain and age group (F_9,882_=2.758, p=0.003), network and age group (F_27,2646_=1.732, p=0.011), and domain and network and age group (F_81,7938_=1.784, p<.001). Follow-up analyses of the 3-way interaction showed that there was an interaction between age and network on negative correlation strength during VOCAB (F_27,2673_=1.593, p=0.027) and SPEED (F_27,2673_=3.377, p<.001) tasks, but not during FLUID (F_27,2673_=1.295, p=0.141) and MEM (F_27,2673_=0.810, p=0.743) tasks. Further, the main effect of age on negative correlation strength was significant for every domain except for MEM (VOCAB: F_3,297_=3.082, p-0.028; SPEED: F_3,297_=6.080, p<.001; FLUID: F_3,297_=6.982, p<.001; MEM: F_3,297_=0.441, p=0.724). The interaction between age and network during VOCAB and SPEED showed that some between-network negative correlations showed weakening with age across both tasks (i.e., Vis and FP networks), but some were specific to one task or the other (i.e., Hand and DAN between-network negative correlations only showed an effect of age during SPEED tasks; See Figure 2, Table 2, Supplementary Tables S1-3).

**3.2 System Segregation.** A 4 (Domain: VOCAB, SPEED, FLUID, MEM) x 2 (Network: Somatomotor vs. Association) x 4 (Age Group: YA, yMA, oMA, OA) MANCOVA revealed a significant main effect of age group (F_3,294_=6.528, p<.001), and a significant interaction between network and domain (F_3,882_=13,412, p<.001) on system segregation. The main effects of domain (F_3,882_=1.631, p=0.181) and network (F_1,294_=3.671, p=0.056), and the interactions among network and age group (F_3,294_=1.695, p=0.168), domain and age group (F_9,882_=0.839, p=0.580), and network and domain and age group (F_9,882_=1.765, p=0.071) were not significant. The main effect of age group showed that younger adults (m=0.118, sd=0.185) showed greater system segregation than older adults (m=-0.013, sd=0.184; mean difference = 0.131, p<.001; no other comparisons significant). The interaction between network and domain showed that system segregation was greater in somatomotor networks during FLUID and MEM tasks relative to VOCAB (FLUID-VOCAB t_301_=2.628, p=0.009; MEM-VOCAB t_301_=2.900, p=0.004) and SPEED (FLUID-SPEED t_301_=3.223, p=0.001; MEM-SPEED t_301_=3.233, p=0.001) tasks, and that system segregation was greater in association networks during VOCAB (t_301_=6.885, p<.001), SPEED (t_301_=5.091, p<.001), and FLUID (t_301_=4.104, p<.001) tasks relative to MEM tasks (See Supplementary Figure S1; VOCAB also showed greater system segregation in association systems relative to FLUID tasks; t_301_=3.452, p=0.001).

**3.3 Graph Theory Metrics of Global Connectivity.** A 4 (Domain: VOCAB, SPEED, FLUID, MEM) x 9 (Threshold: 2-10%) x 4 (Age Group: YA, yMA, oMA, OA) MANCOVA revealed significant main effects of domain (F_3,882_=269.141, p<.001) and threshold (F_8,2352_=9530.037, p<.001) on global efficiency. Further, there were significant interactions among domain and age group (F_9,882_=2.064, p=0.030) and domain and threshold (F_24,7056_=79.106, p<.001). The main effect of age group (F_3,294_=2.115, p=0.098), and the interactions among threshold and age group (F_24,2352_=0.477, p=0.985) and domain and threshold and age group (F_72,7056_=0.901, p=0.710) were not significant. The main effect of domain showed that global efficiency was highest during VOCAB tasks, followed by MEM, SPEED, and FLUID (all significantly different, p<.001). The main effect of threshold showed that global efficiency increases with threshold (all thresholds significantly different, p<.001). The interaction between domain and threshold showed that the effect of domain on global efficiency differed by threshold (see Supplementary Figure 2a). The interaction between age and domain was driven by YAs showing higher levels of global efficiency than oMAs (mean difference=0.006, p=0.015) and OAs (mean difference=0.007, p=0.003) during FLUID tasks (F_3,297_=4.718, p=0.003), but no effect on age on global efficiency during VOCAB (F_3,297_=0.731, p=0.534), SPEED (F_3,297_=1.682, p=0.171), or MEM (F_3,297_=1.336, p=0.263) tasks (see Supplementary Figure 2b).

A 4 (Domain: VOCAB, SPEED, FLUID, MEM) x 9 (Threshold: 2-10%) x 4 (Age Group: YA, yMA, oMA, OA) MANCOVA revealed significant main effects of domain (F_3,882_=12.475, p<.001), threshold (F_8,2352_=478.160, p<.001), and age group (F_3,294_=8.927, p<.001) on modularity. Additionally, there were significant interactions among domain and age group (F_9,882_=2.791, p=0.003), threshold and age group (F_24,2352_=2.896, p<.001), and domain and threshold (F_24,7056_=101.308, p<.001). The interaction among domain, threshold, and age group was not significant (F_72,7056_=0.578, p=0.998). The main effect of domain showed that, on average, modularity was highest during VOCAB tasks (greater than all other domains, all Bonferroni-corrected p-values<0.010), followed by FLUID tasks (greater than SPEED, p=0.011; and MEM, p=0.001). The main effect of threshold showed that modularity decreases with threshold (all thresholds significantly different, p<.001). The main effect of age showed that YAs (mean difference=0.019, p<.001) and yMAs (mean difference=0.025, p=0.004) had greater modularity than OAs, and YAs had greater modularity than oMAs (mean difference=0.019, p=0.047). The interaction between domain and threshold showed that the effect of domain on modularity differed by threshold (see Supplementary Figure 3a). The interaction between age and domain showed that there was a significant effect of age on modularity only during SPEED and FLUID tasks (see Supplementary Figure 3b). Finally, the interaction between age group and threshold showed that the effect of age group on modularity differed by threshold (see Supplementary Figure 3c).

Supplementary Table 1 (S1)

Post-hoc test results for the main effect of age at each post-hoc contrast for both positive and negative correlation values computed during performance of VOCAB tasks. Values represent the mean difference (p-values in parentheses) for each age group pairing; significant mean differences are bolded.

|  | **YA vs. yMA** | **YA vs. oMA** | **YA vs. OA** | **yMA vs. oMA** | **yMA vs. OA** | **oMA vs. OA** |
| --- | --- | --- | --- | --- | --- | --- |
| **VOCAB - Positive Correlations** | | | | | | |
| ***3-way MANCOVA by Domain*** | | | | | | |
| Main Effect of Age | 0.013 (0.101) | **0.019 (0.001)** | **0.021 (<.001)** | 0.006 (>.999) | 0.009 (0.499) | 0.003 (>.999) |
| ***2-way MANCOVA by Domain and Direction*** | | | | | | |
| *Within-Network* |  |  |  |  |  |  |
| Main Effect of Age | **0.017 (0.025)** | **0.025 (<.001)** | **0.029 (<.001)** | 0.008 (0.906) | 0.013 (0.154) | 0.005 (>.999) |
| *Between-Network* |  |  |  |  |  |  |
| Main Effect of Age | 0.009 (0.527) | **0.014 (0.034)** | **0.014 (0.023)** | 0.004 (>.999) | 0.005 (>.999) | 0.001 (>.999) |
| **1-way post-hoc ANOVAs** | | | | | | |
| *Within-Network* |  |  |  |  |  |  |
| Hand | 0.029 (0.097) | 0.015 (>.999) | **0.032 (0.025)** | -0.015 (>.999) | 0.003 (>.999) | 0.017 (0.619) |
| Vis | 0.032 (0.098) | **0.076 (<.001)** | **0.067 (<.001)** | **0.043 (0.005)** | 0.034 (0.051) | -0.009 (>.999) |
| Mouth | 0.039 (0.050) | **0.067 (<.001)** | **0.064 (<.001)** | 0.028 (0.297) | 0.025 (0.441) | -0.002 (>.999) |
| Aud | 0.013 (>.999) | 0.014 (0.751) | **0.034 (0.002)** | 0.001 (>.999) | 0.020 (0.227) | 0.019 (0.169) |
| DMN | 0.020 (0.507) | 0.023 (0.199) | **0.037 (0.003)** | 0.003 (>.999) | 0.017 (0.722) | 0.015 (0.873) |
| FP | *n/a* | | | | | |
| VAN | *n/a* | | | | | |
| CO | 0.019 (0.663) | 0.023 (0.195) | **0.036 (0.007)** | 0.004 (>.999) | 0.017 (0.852) | 0.013 (>.999) |
| DAN | *n/a* | | | | | |
| Sal | *n/a* | | | | | |
| *Between-Network* |  |  |  |  |  |  |
| Hand | *n/a* | | | | | |
| Vis | *n/a* | | | | | |
| Mouth | 0.014 (0.693) | **0.024 (0.023)** | 0.021 (0.078) | 0.010 (>.999) | 0.007 (>.999) | -0.003 (>.999) |
| Aud | 0.012 (0.479) | **0.019 (0.017)** | **0.023 (0.002)** | 0.007 (>.999) | 0.011 (0.691) | 0.004 (>.999) |
| DMN | 0.008 (>.999) | **0.015 (0.030)** | **0.016 (0.014)** | 0.007 (>.999) | 0.009 (0.731) | 0.001 (>.999) |
| FP | *n/a* | | | | | |
| VAN | 0.005 (>.999) | **0.019 (0.030)** | 0.015 (0.145) | 0.014 (0.264) | 0.011 (0.824) | -0.004 (>.999) |
| CO | *n/a* | | | | | |
| DAN | *n/a* | | | | | |
| Sal | *n/a* | | | | | |
| **VOCAB - Negative Correlations** | | | | | | |
| ***2-way MANCOVA by Domain*** | | | | | | |
| Main Effect of Age | -0.008 (0.676) | -0.004 (>.999) | **-0.013 (0.024)** | 0.004 (>.999) | -0.005 (>.999) | -0.009 (0.223) |
| **1-way post-hoc ANOVAs** | | | | | | |
| *Between-Network* |  |  |  |  |  |  |
| Hand | *n/a* | | | | | |
| Vis | -0.02 (0.092) | -0.009 (>.999) | **-0.024 (0.008)** | 0.011 (>.999) | -0.004 (>.999) | -0.015 (0.202) |
| Mouth | *n/a* | | | | | |
| Aud | *n/a* | | | | | |
| DMN | -0.001 (>.999) | -0.003 (>.999) | **-0.013 (0.042)** | -0.002 (>.999) | -0.013 (0.084) | -0.011 (0.152) |
| FP | -0.009 (0.845) | -0.004 (>.999) | **-0.019 (0.005)** | 0.005 (>.999) | -0.010 (0.590) | **-0.015 (0.041)** |
| VAN | -0.010 (>.999) | -0.014 (0.214) | **-0.024 (0.002)** | -0.004 (>.999) | -0.014 (0.226) | -0.010 (0.570) |
| CO | *n/a* | | | | | |
| DAN | *n/a* | | | | | |
| Sal | *n/a* | | | | | |

Supplementary Table 2 (S2)

Post-hoc test results for the main effect of age at each post-hoc contrast for both positive and negative correlation values computed during performance of SPEED tasks. Values represent the mean difference (p-values in parentheses) for each age group pairing; significant mean differences are bolded.

|  | **YA vs. yMA** | **YA vs. oMA** | **YA vs. OA** | **yMA vs. oMA** | **yMA vs. OA** | **oMA vs. OA** |
| --- | --- | --- | --- | --- | --- | --- |
| **SPEED - Positive Correlations** | | | | | | |
| ***3-way MANCOVA by Domain*** | | | | | | |
| Main Effect of Age | 0.008 (0.550) | 0.009 (0.345) | **0.015 (0.010)** | 0.000 (>.999) | 0.006 (>.999) | 0.006 (>.999) |
| ***2-way MANCOVA by Domain and Direction*** | | | | | | |
| *Within-Network* |  |  |  |  |  |  |
| Main Effect of Age | 0.011 (0.277) | **0.015 (0.018)** | **0.022 (<.001)** | 0.004 (>.999) | 0.011 (0.268) | 0.007 (0.900) |
| *Between-Network* |  |  |  |  |  |  |
| Main Effect of Age | *n/a* | | | | | |
| **1-way post-hoc ANOVAs** | | | | | | |
| *Within-Network* |  |  |  |  |  |  |
| Hand | *n/a* | | | | | |
| Vis | -0.012 (>.999) | 0.032 (0.087) | 0.019 (0.918) | **0.044 (0.009)** | 0.031 (0.157) | -0.013 (>.999) |
| Mouth | **0.042 (0.008)** | **0.050 (<.001)** | **0.059 (<.001)** | 0.008 (>.999) | 0.017 (>.999) | 0.009 (>.999) |
| Aud | 0.008 (>.999) | 0.018 (0.348) | **0.038 (0.001)** | 0.010 (>.999) | **0.029 (0.019)** | 0.020 (0.177) |
| DMN | **0.048 (<.001)** | 0.023 (0.196) | **0.046 (<.001)** | -0.025 (0.18) | -0.002 (>.999) | 0.023 (0.161) |
| FP | *n/a* | | | | | |
| VAN | *n/a* | | | | | |
| CO | *n/a* | | | | | |
| DAN | *n/a* | | | | | |
| Sal | *n/a* | | | | | |
| *Between-Network* |  |  |  |  |  |  |
| Hand | *n/a* | | | | | |
| Vis | *n/a* | | | | | |
| Mouth | 0.014 (0.477) | 0.006 (>.999) | **0.020 (0.034)** | -0.008 (>.999) | 0.006 (>.999) | 0.014 (0.228) |
| Aud | *n/a* | | | | | |
| DMN | *n/a* | | | | | |
| FP | *n/a* | | | | | |
| VAN | *n/a* | | | | | |
| CO | *n/a* | | | | | |
| DAN | *n/a* | | | | | |
| Sal | *n/a* | | | | | |
| **SPEED - Negative Correlations** | | | | | | |
| ***2-way MANCOVA by Domain*** | | | | | | |
| Main Effect of Age | -0.009 (0.464) | 0.001 (>.999) | **-0.015 (0.007)** | 0.010 (0.199) | -0.006 (>.999) | **-0.017 (0.001)** |
| **1-way post-hoc ANOVAs** | | | | | | |
| *Between-Network* |  |  |  |  |  |  |
| Hand | -0.009 (0.965) | 0.007 (>.999) | **-0.019 (0.007)** | 0.015 (0.062) | -0.010 (0.602) | **-0.025 (<.001)** |
| Vis | **-0.029 (0.003)** | -0.017 (0.153) | **-0.045 (<.001)** | 0.012 (0.778) | -0.015 (0.354) | **-0.028 (0.001)** |
| Mouth | *n/a* | | | | | |
| Aud | *n/a* | | | | | |
| DMN | *n/a* | | | | | |
| FP | -0.012 (0.312) | 0.005 (>.999) | **-0.016 (0.023)** | **0.017 (0.022)** | -0.004 (>.999) | **-0.021 (<.001)** |
| VAN | *n/a* | | | | | |
| CO | *n/a* | | | | | |
| DAN | -0.020 (0.106) | -0.006 (>.999) | **-0.032 (<.001)** | 0.014 (0.486) | -0.012 (0.955) | **-0.026 (0.004)** |
| Sal | -0.001 (>.999) | 0.010 (0.299) | -0.004 (>.999) | 0.010 (0.272) | -0.004 (>.999) | **-0.014 (0.020)** |

Supplementary Table 3 (S3)

Post-hoc test results for the main effect of age at each post-hoc contrast for both positive and negative correlation values computed during performance of FLUID tasks. Values represent the mean difference (p-values in parentheses) for each age group pairing; significant mean differences are bolded.

|  | **YA vs. yMA** | **YA vs. oMA** | **YA vs. OA** | **yMA vs. oMA** | **yMA vs. OA** | **oMA vs. OA** |
| --- | --- | --- | --- | --- | --- | --- |
| **FLUID - Positive Correlations** | | | | | | |
| ***3-way MANCOVA by Domain*** | | | | | | |
| Main Effect of Age | -0.001 (>.999) | **0.019 (<.001)** | **0.025 (<.001)** | **0.020 (<.001)** | **0.026 (<.001)** | 0.006 (>.999) |
| ***2-way MANCOVA by Domain and Direction*** | | | | | | |
| *Within-Network* |  |  |  |  |  |  |
| Main Effect of Age | 0.003 (>.999) | **0.026 (<.001)** | **0.032 (<.001)** | **0.023 (<.001)** | **0.029 (<.001)** | 0.006 (>.999) |
| *Between-Network* |  |  |  |  |  |  |
| Main Effect of Age | -0.005 (>.999) | 0.012 (0.065) | **0.017 (0.002)** | **0.017 (0.003)** | **0.022 (<.001)** | 0.005 (>.999) |
| **1-way post-hoc ANOVAs** | | | | | | |
| *Within-Network* |  |  |  |  |  |  |
| Hand | *n/a* | | | | | |
| Vis | 0.021 (0.407) | **0.058 (<.001)** | **0.067 (<.001)** | **0.037 (0.004)** | **0.046 (<.001)** | 0.009 (>.999) |
| Mouth | 0.011 (>.999) | **0.041 (0.005)** | **0.063 (<.001)** | 0.030 (0.128) | **0.052 (<.001)** | 0.022 (0.376) |
| Aud | -0.008 (>.999) | **0.037 (0.001)** | **0.053 (<.001)** | **0.045 (<.001)** | **0.061 (<.001)** | 0.015 (0.587) |
| DMN | *n/a* | | | | | |
| FP | *n/a* | | | | | |
| VAN | *n/a* | | | | | |
| CO | 0.004 (>.999) | **0.032 (0.010)** | **0.043 (<.001)** | 0.028 (0.061) | **0.038 (0.003)** | 0.010 (>.999) |
| DAN | 0.019 (>.999) | 0.038 (0.120) | **0.044 (0.042)** | 0.019 (>.999) | 0.025 (0.858) | 0.006 (>.999) |
| Sal | *n/a* | | | | | |
| *Between-Network* |  |  |  |  |  |  |
| Hand | -0.002 (>.999) | 0.013 (0.133) | **0.018 (0.010)** | 0.015 (0.079) | **0.020 (0.006)** | 0.005 (>.999) |
| Vis | *n/a* | | | | | |
| Mouth | -0.010 (0.902) | 0.016 (0.115) | 0.016 (0.104) | **0.026 (0.001)** | **0.026 (0.001)** | 0.000 (>.999) |
| Aud | -0.001 (>.999) | **0.024 (0.001)** | **0.032 (<.001)** | **0.025 (0.001)** | **0.033 (<.001)** | 0.007 (>.999) |
| DMN | 0.000 (>.999) | **0.013 (0.020)** | **0.017 (0.001)** | **0.013 (0.024)** | **0.018 (0.001)** | 0.004 (>.999) |
| FP | *n/a* | | | | | |
| VAN | 0.007 (>.999) | **0.021 (0.008)** | **0.028 (<.001)** | 0.014 (0.290) | **0.021 (0.016)** | 0.007 (>.999) |
| CO | -0.006 (>.999) | **0.018 (0.006)** | **0.024 (<.001)** | **0.024 (<.001)** | **0.030 (<.001)** | 0.006 (>.999) |
| DAN | *n/a* | | | | | |
| Sal | -0.009 (0.703) | 0.010 (0.371) | **0.019 (0.003)** | **0.019 (0.004)** | **0.028 (<.001)** | 0.009 (0.465) |
| **FLUID - Negative Correlations** | | | | | | |
| ***2-way MANCOVA by Domain*** | | | | | | |
| Main Effect of Age | 0.001 (>.999) | -0.009 (0.320) | **-0.017 (0.001)** | -0.009 (0.267) | **-0.018 (0.001)** | -0.009 (0.232) |
| **1-way post-hoc ANOVAs** | | | | | | |
| *Between-Network* |  |  |  |  |  |  |
| Hand | *n/a* | | | | | |
| Vis | *n/a* | | | | | |
| Mouth | *n/a* | | | | | |
| Aud | *n/a* | | | | | |
| DMN | *n/a* | | | | | |
| FP | *n/a* | | | | | |
| VAN | *n/a* | | | | | |
| CO | *n/a* | | | | | |
| DAN | *n/a* | | | | | |
| Sal | *n/a* | | | | | |

Supplementary Table 4 (S4)

Post-hoc test results for the main effect of age at each post-hoc contrast for both positive and negative correlation values computed during performance of MEM tasks. Values represent the mean difference (p-values in parentheses) for each age group pairing; significant mean differences are bolded.

|  | **YA vs. yMA** | **YA vs. oMA** | **YA vs. OA** | **yMA vs. oMA** | **yMA vs. OA** | **oMA vs. OA** |
| --- | --- | --- | --- | --- | --- | --- |
| **MEM - Positive Correlations** | | | | | | |
| ***3-way MANCOVA by Domain*** | | | | | | |
| Main Effect of Age | 0.002 (>.999) | **0.011 (0.049)** | **0.013 (0.024)** | 0.010 (0.177) | 0.011 (0.096) | 0.001 (>.999) |
| ***2-way MANCOVA by Domain and Direction*** | | | | | | |
| *Within-Network* |  |  |  |  |  |  |
| Main Effect of Age | 0.004 (>.999) | **0.016 (0.003)** | **0.018 (0.001)** | **0.013 (0.046)** | **0.015 (0.014)** | 0.002 (>.999) |
| *Between-Network* |  |  |  |  |  |  |
| Main Effect of Age | *n/a* | | | | | |
| **1-way post-hoc ANOVAs** | | | | | | |
| *Within-Network* |  |  |  |  |  |  |
| Hand | *n/a* | | | | | |
| Vis | -0.002 (>.999) | 0.018 (0.519) | **0.037 (0.002)** | 0.020 (0.418) | **0.039 (0.002)** | 0.019 (0.297) |
| Mouth | -0.001 (>.999) | 0.012 (>.999) | 0.036 (0.052) | 0.012 (>.999) | 0.036 (0.067) | 0.024 (0.391) |
| Aud | -0.004 (>.999) | 0.018 (0.311) | **0.042 (<.001)** | 0.022 (0.150) | 0.046 (<.001) | 0.024 (0.056) |
| DMN | *n/a* | | | | | |
| FP | *n/a* | | | | | |
| VAN | *n/a* | | | | | |
| CO | 0.017 (0.792) | **0.035 (0.004)** | **0.030 (0.022)** | 0.018 (0.547) | 0.013 (>.999) | -0.005 (>.999) |
| DAN | *n/a* | | | | | |
| Sal | *n/a* | | | | | |
| *Between-Network* |  |  |  |  |  |  |
| Hand | *n/a* | | | | | |
| Vis | *n/a* | | | | | |
| Mouth | *n/a* | | | | | |
| Aud | *n/a* | | | | | |
| DMN | *n/a* | | | | | |
| FP | *n/a* | | | | | |
| VAN | *n/a* | | | | | |
| CO | *n/a* | | | | | |
| DAN | *n/a* | | | | | |
| Sal | *n/a* | | | | | |
| **MEM - Negative Correlations** | | | | | | |
| ***2-way MANCOVA by Domain*** | | | | | | |
| Main Effect of Age | *n/a* | | | | | |
| **1-way post-hoc ANOVAs** | | | | | | |
| *Between-Network* |  |  |  |  |  |  |
| Hand | *n/a* | | | | | |
| Vis | *n/a* | | | | | |
| Mouth | *n/a* | | | | | |
| Aud | *n/a* | | | | | |
| DMN | *n/a* | | | | | |
| FP | *n/a* | | | | | |
| VAN | *n/a* | | | | | |
| CO | *n/a* | | | | | |
| DAN | *n/a* | | | | | |
| Sal | *n/a* | | | | | |

Supplementary Figure 1 (S1)

Average association (Assoc) and somatomotor (Somato) system segregation across all participants for each task domain. Asterisks reflect significance of the differences within each network grouping (*p<.05, **p<.01, ***p<.001; all Bonferroni corrected).


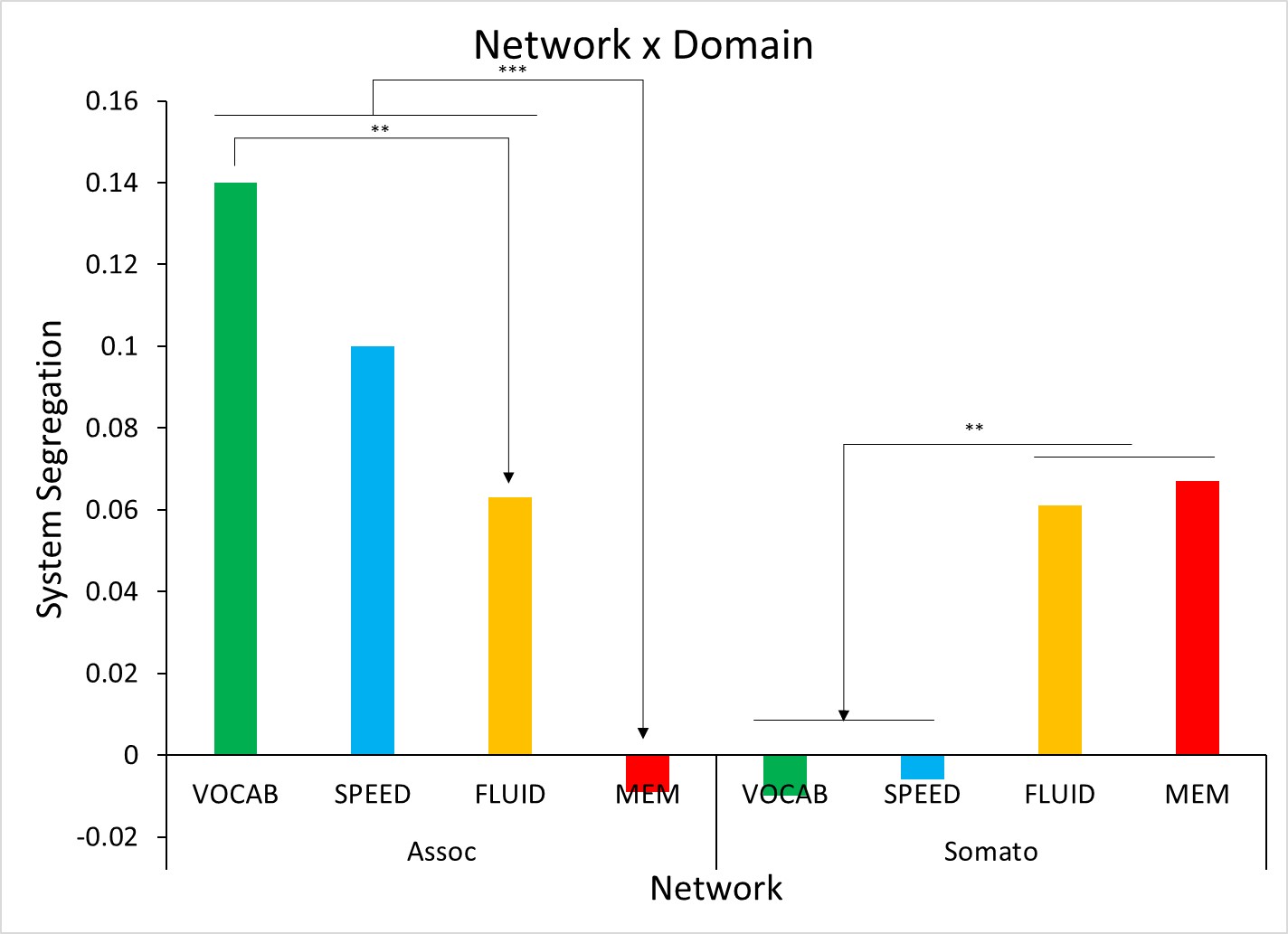


Supplementary Figure 2 (S2)

Average global efficiency as a function of task domain and threshold, and as a function of task domain and age group. In panel (a), letters reflect differences among the four task domains at each threshold (a: MEM>VOCAB>SPEED=FLUID; b: MEM>VOCAB>SPEED>FLUID; c: MEM=VOCAB>SPEED>FLUID; d: VOCAB>MEM>SPEED>FLUID; e: VOCAB>MEM=SPEED>FLUID); in panel (b), asterisks reflect significance of the differences between age groups within each domain (*p<.05, **p<.01, ***p<.001; all Bonferroni corrected).


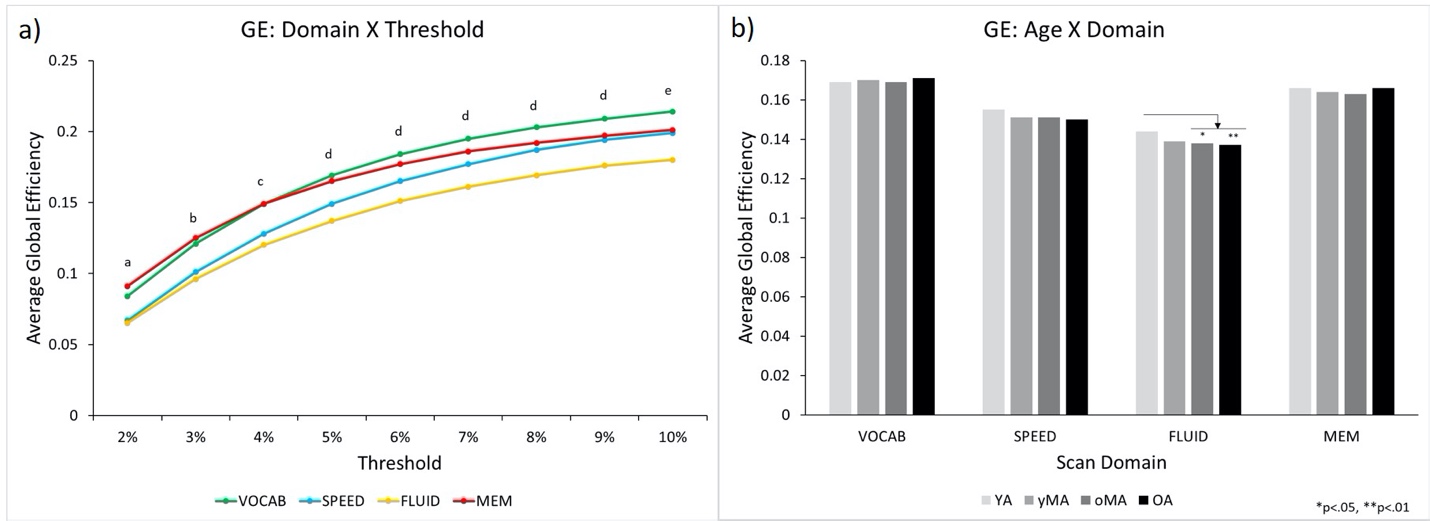


Supplementary Figure 3 (S3)

Average modularity (Mod) as a function of task domain and threshold, as a function of task domain and age group, and as a function of age group and threshold. In panel (a), letters reflect differences among the four task domains at each threshold (u: VOCAB=MEM>SPEED>FLUID; v: VOCAB>all, FLUID/MEM>SPEED; w: VOCAB>all, FLUID>SPEED; x: VOCAB>all, FLUID>MEM/SPEED; y: VOCAB>SPEED/MEM, FLUID>MEM; z: all>MEM, VOCAB>SPEED); in panel (b), asterisks reflect significance of the differences between age groups within each domain (*p<.05, **p<.01, ***p<.001; all Bonferroni corrected); in panel (c), symbols reflect differences among the four age groups at each threshold (%: YA>oMA/OA, yMA>OA; ^: YA/yMA>OA; $: all>OA)


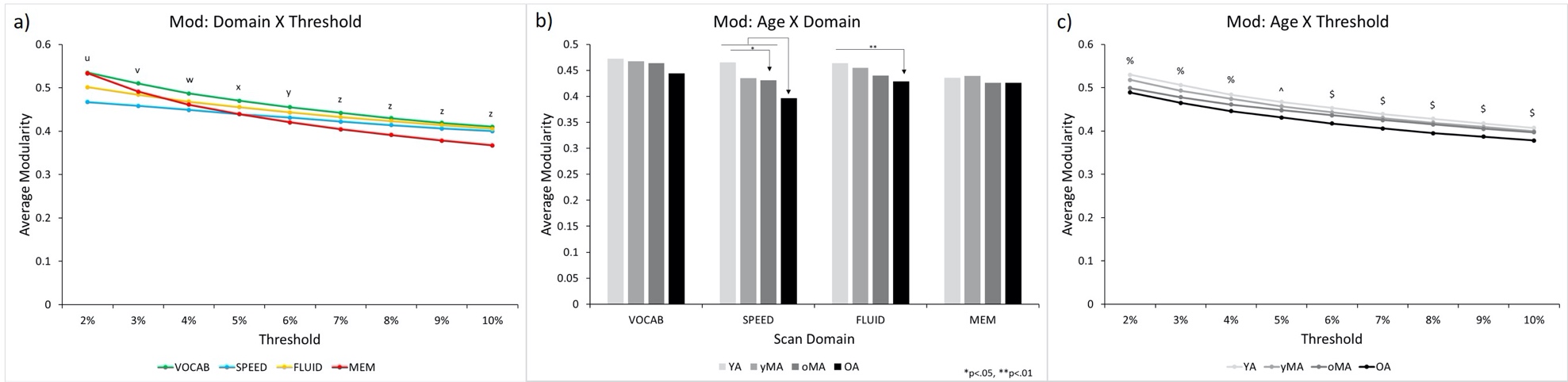

Supplement: Supplementary file 1 — Supplementary Material [file BRB3-11-e01954-s001.docx]
